
JOURNAL INFORMATION
==============================
NLM Title Abbreviation: JBRA Assist Reprod
Journal ID: JBRA Assist Reprod
NLM Journal ID: 101684552
Journal ID: jbra

JBRA Assisted Reproduction

ISSN: 1517-5693
EISSN: 1518-0557
Publisher: Brazilian Society of Assisted Reproduction

ARTICLE INFORMATION
==============================
PMCID: PMC7558895
PMID: 33021752
DOI: 10.5935/1518-0557.20200073
Article version: 1
Subjects: Poster Presentations

Abstracts of the 24th Annual Congress of the SBRA, 2020 (Virtual Meeting)

Publication date: 2020 Oct-Dec
Print publication date: 2020 Oct-Dec
Volume: 24
Issue: 4
First page: 525
Last page: 550
License: This is an Open Access article distributed under the terms of the Creative Commons Attribution License, which permits unrestricted use, distribution, and reproduction in any medium, provided the original work is properly cited.
License URL: https://creativecommons.org/licenses/by/4.0/

==============================


P-01. Cumulation of embryos in IVF cycles with PGT-M as a strategy for family planning in a couple with Osteogenesis Imperfecta Type IV

Ceschin Ianaê 1 2
Bandeira Gabriel 2
Nishikawa Lucileine 1
Ceschin Nathan 1
Carvalho Cristina 3
Riboldi Marcia 3
Ali Taccyanna 3
Santander Paulina 1
Ceschin Alvaro 1
1 Feliccità Instituto de Fertilidade - Curitiba, Paraná, Brasil
2 Centro de Estudos sobre o Genoma Humano e Células-Tronco (CEGH-CEL), Departamento de Biologia Evolutiva, Instituto de Biociências - Universidade de São Paulo, São Paulo, Brasil.
3 Laboratório Igenomix - Laboratório de Genética e Medicina Reprodutiva - São Paulo, Brasil.

P-02. Relationship between age and chromosomal ploidy of blastocysts analyzed by noninvasive preimplantation genetic testing for aneuploidies (niPGT-A)

Vagnini L.D. 1
Petersen C.G. 1 2
Renzi A. 1
Dieamant F. 2
Oliveira J.B.A. 2
Oliani A.H. 3
Canas M.C.T. 1
Nakano R. 4
Almodin C.G. 5
Marcondes C. 6
Ceschin A. 7
Amaral A. 8
Soares J.B. 9
Lopes J. 10
Franco A.C. 11
Franco Jr. J.G. 1 2
1 Paulista Center for Diagnosis Research and Training, Ribeirao Preto - CPDP, Brazil.
2 Centre for Human Reproduction Prof Franco Jr, Ribeirao Preto, Brazil.
3 São José do Rio Preto School of Medicine FAMERP, Sao Jose do Rio Preto, Brazil.
4 Ferticlin Human Fertility Clinic, Sao Paulo, Brazil.
5 Materbaby, Maringa, Brazil.
6 Santista Nucleus of Human Reproduction, Santos, Brazil.
7 Feliccita Fertility Institute, Curitiba, Brazil.
8 Genesis Human Reproduction Assistance Center, Brasília, Brazil.
9 Alpha Project-Alliance of Assisted Fertilization Laboratories, São Paulo, Brazil.
10 Cenafert, Salvador, Brazil.
11 Embryolife, São José dos Campos, Brazil

P-03. Quality of online information provided by fertility clinic websites: compliance with Brazilian Medical Council (CFM) and American Society for Reproductive Medicine (ASRM) guidelines

**Objective**: Osteogenesis Imperfecta (OI) is a disease characterized by fragile bones and a decrease in bone mass. There are approximately nineteen types of OI differing from each other by morbidity, mortality and causative mutation. The OI Type IV occurs in most cases of deleterious variant in heterozygosis (autosomal dominant) in the COL1A1 or COL1A2 genes. Those genes are responsible for the production of procollagen chains that are part of type I collagen molecule. Patients with OI type IV have a high risk (50%) of transmitting the pathogenic variant to their offspring being recommendable genetic counseling to discuss family planning alternatives. The use of assisted human reproduction techniques followed by analysis of Pre-implantation Genetic Test for Monogenic Diseases (PGT-M) in embryos has proven to be an effective alternative to reduce the risk of recurrence. Objective: Family planning with In Vitro Fertilization (IVF) cycles and cumulation of frozen embryos followed by PGT-M as an alternative to reduce the risk of transmission of the pathogenic variant that causes OI Type IV.
**Methods**: The case reported a couple in which the male patient has a diagnosis of OI Type IV caused by a deleterious variant in COL1A2. Informative study was conducted with the carrier and his wife before the IVF cycle and the PGT-M. The previous test was designed to find the informative polymorphic Short Tandem Repeats (STR) markers linked to mutations or genomic regions. For the IVF treatment three cycles were made with the same stimulation protocol. It was used clomiphene citrate, gonadotropin, antagonist GnRh and trigger with agonist GnRh. In the first cycle, six embryos were produced and frozen on the third day of development (D3). In the following cycle three embryos had vitrified in the same development day. In the third cycle six embryos were developed and after they reached cleavage state (D3), all other embryos, from the previous cycles, were thawed, survived the technique and developed until the blastocyst stage. From the total fifteen embryos in the cleavage stage, nine evolved to the blastocyst stage. The PGT-M was performed at all blastocysts followed by embryo cryopreservation. Regarding the genetic analysis of embryo cells, for those samples that the deleterious variant in COL1A2 was not found, the research advanced with Pre-implantation Genetic Test for Aneuploidy (PGT-A). The antagonist protocol used for the endometrial preparation in the embryo thaw cycle, from the third day of menstrual flow progressive doses of estradiol were used until the appropriate thickness and pattern was reached. It was administered 10.000 UI of urinary HCG subcutaneous. After 48 hours, 75 mg of intramuscular progesterone was started. On the sixth day of administration, the euploid embryo without the mutation searched was thawed and transferred to the uterine cavity.
**Results**: In this cohort of nine embryos, there were five embryos with COL1A2 mutation present. From the four embryos without the mutation, three of them were aneuploids. One embryo did not present the haplotype with the mutation in COL1A2 and was euploid. This embryo was thawed and followed by transfer and resulted in an unaffected live birth.
**Conclusion**: This article evidences an IVF cycle with a multidisciplinary approach with genetic counseling, serial ovarian stimulation, cumulative IVF cycles with embryo freezing, PGT-M, PGT-A and cycle of thawing can be a viable treatment to reduce recurrence risk of autosomal-dominant disease (50%).

**Objective:** To assess the relationship between human blastocyst chromosomal ploidy established by niPGT-A and increasing age.
**Methods:** This is a multicenter prospective study performed by ten assisted reproduction centers after training and validation process of the embryologists to use niPGT-A. A total of 94 couples with indication for niPGT-A due to increase maternal age, male factor, repeated implantation failures, recurrent abortion or because they requested niPGT-A were included in this work. All couples had no karyotype abnormalities. After ICSI, embryos were cultured until blastocyst stage using one or two step culture systems, single or sequential media respectively, at 37°C in an atmosphere of 6-7% $CO_2$ and 5-20% $O_2$ incubators. On day 3, cleavage embryos were re-evaluated to complete removal of the cumulus cells. Embryos were then cultured in an individual well with 20 µl of medium in GPS dishware under oil and cultured until they reach blastocyst stage. The blastocysts were vitrified and stored in liquid nitrogen. After that, the spent blastocyst culture medium (20µl) was transferred to a PCR tube and sent to the genetic analysis laboratory, where it was stored at -80°C until sequencing. A total of 220 samples of spent blastocyst culture medium were collected on the 5th/6th day. Cell-free DNA secreted on culture medium was amplified using NICS Sample Preparation Kit (Yikon Genomics), based on MALBAC technology. After whole genome amplification, the DNA was measured using a Qubit 2.0 fluorometer and subjected to next generation sequencing (NGS) using Illumina MiSeq® platform. The results were analyzed by ChromGo® software (Yikon Genomics).

**Results:** The mean age of the patients was 38±4.08 with an interval of 20-44 years. The euploid was diagnosed in 36.4% (80/220) of cases, aneuploidy in 31.3% (69/220), and mosaicism in 32.3% (71/220; with ≥60% aneuploidy) of blastocysts. Mosaic values ranged from 29.8% to 33.8% in different age groups (Table 1). Individually, the most frequent chromosomal abnormality was XXY (Klinefelter Syndrome) occurring in 18 cases, followed by chromosome 21 (trisomy/monosomy) in 8 cases (Table 2). niPGT-A data showed an ≥60% incidence of aneuploid cells in all cases of chromosomal mosaicism (n=71).

**Conclusion:** A high degree of mosaicism with aneuploidy cells was detected and some hypotheses were suggested for this data (sensitivity of niPGT-A in detecting the phenomenon of self-correction of chromosomal abnormalities). However, it did not vary remarkably with age. On the other hand, euploidy levels had a negative correlation with age and aneuploidy levels had a positive relationship. This is the first report in the literature to relate chromosomal ploidy in blastocysts using niPGT-A and increasing patient age.

**M.M. Carneiro[1,2], C.N. Koga[1], M.C.L. Mussi[1], P.F. Fradico[1], M.C.F. Ferreira[1,2]**

**Objective**: To evaluate 11th SisEmbrio-registered fertility clinic websites in the State of Minas Gerais (MG), Brazil, regarding their compliance with the 2004 American Society for Reproductive Medicine (ASRM) and the Brazilian Medical Council (CFM) guidelines for advertising and to survey the general features of their websites and social media.

**Methods:** We performed a cross-sectional online evaluation to obtain data on the registered clinics websites using criteria based on 2004 ASRM guidelines for advertising (Success rates published; Presence of data to support success rate; Comparison marketing; Live birth rate reported; Method of calculating live birth rate provided; Live birth rate reported for time period given; Success rates on based on age; Success rates based on diagnosis; Experimental/ Investigational nature of procedure defined) and compliance with CFM guidelines (practice director visible on the website with respective council number; no information on costs of treatments, no photos of patients shown nor success stories with paient identification) for advertisement. The general characteristics of fertility clinics websites registered in the 11th Report of the National Embryo Production System platform (SisEmbrio 2017) and their social media in Minas Gerais.

**Results:** Available online data from 19 fertility clinics and 4 branches registered in 11th SisEmbrio were analyzed, revealing 18 private and one public clinic. Ten clinics are in located in the capital and 12 in the interior of the state, four of which are branches. Regarding the recommendations established by the CFM Medical Advertising Manual, only eight presented the practice director visible on the website with their respective council number. Patients' photos were displayed by 14 clinics in their websites, and by 8 in their social networks. Fifteen clinics reported success stories and testimonials from patients about their experience with the clinic and/or treatment. None of the websites displayed costs nor offered experimental procedures and exclusive techniques. Number of doctors of each clinic ranged from one to 10 and only four clinics had andrology and genetics specialists. Eighteen websites displayed the curriculum of the medical team, 14 presented photos of the team and only four and published scientific articles. Thirteen centers reported success rates but these were not the clinic's own rates, nor did they disclose rates of live births per treatment. Regarding the use of social networks, 12 reported a WhatsApp number, 15 had Facebook pages (211 to 1354 followers), 14 exhibited profiles on Instagram (1232 to 1980 followers), and one on LinkedIn, 5 had channels on YouTube and one had a Podcast. Twenty-one clinics offered explanations about the procedures to patients and 16 had a "virtual tour" of the clinic's facilities. Among the treatments offered we found: IVF (n = 21), IUI (n = 20), programmed intercourse (n = 19), preservation of fertility (social, n = 20; oncologic, n = 18), PGT (n = 14), oocyte donation (n = 8), uterus surrogacy (n = 9) and semen donation (n = 4).

**Conclusion**: Online Information provided by fertility clinics in Minas Gerais is heterogeneous. A significant proportion of the SisEmbrio-registered fertility clinics websites do not follow some aspect of ASRM and CFM guidelines for advertising. As websites and social media are widely used by patients to obtain health information, increased dissemination and awareness of the guidelines is highly recommended.

## P-04. Successful lyophilization of human spermatozoa confirmed by flow cytometry analysis of membrane integrity

**S. Geber[1,2], C.Q. Giraldo[1], R.l. Bossi[2], A.T. Carvalho[3], S.O.A. Fernandes[4], M. Sampaio[2]**

**Objective**: Cryopreservation of human spermatozoa is a well-established technique that have been used in the last decades for several indications. The use of liquid nitrogen, however, is costly, requires continuous monitoring and large storage spaces and is associated with the risk of cross-contamination between samples. Lyophilization is widely used for dehydration of food, pharmaceuticals, biotechnology products, vaccines, biological materials and diagnostics, it could be a more practical and cost-effective alternative for sperm preservation. Therefore, our aim was to assess membrane integrity of lyophilized human spermatozoa using flow cytometry.

**Methods**: The study was approved by the Research Ethics Committee of the Universidade Federal de Minas Gerais (COEP/UFMG-No 743.984) and all participants read and signed informed consent from. Human normozoospermic semen samples were collected from 32 healthy donors. Samples were divided into two aliquots: one for cryopreservation/lyophilization and the other for cryopreservation (control). After 2 months, samples were thawed or rehydrated and analysed for membrane integrity using flow cytometry. Cryopreservation was performed through a slow addition of a cryoprotectant medium. All cryopreserved samples were kept in cryotubes and placed in the liquid nitrogen vapor for 30 minutes and plunged in liquid nitrogen. Thaw was performed in water bath, suspension was flushed Gamete Buffer, centrifugated and the pellet was resuspended in Saline Phosphate Buffer. Lyophilization started with the addition buffer and EDTA. Vials were placed in a lyophilization machine vacuumed at 0.37 Mbar pressure for 19.5 hours. The lyophilized samples were stored at 4°C for 2 months. Subsequently, were resuspended in water and analysed. Flow cytometry was performed using Annexin V-FLUOS Staining apoptosis detection kit and Propidium Iodide to differentiate apoptosis and necrosis. Result obtained was classified according to the type of mark presented: late apoptosis (Q1 - positive IP negative AV), necrosis (Q2 - double positive), initial apoptosis (Q3 - positive AV negative IP), preserved cell membrane integrity (Q4 - double negative).

**Results**: Age ranged from 31 and 49 years (mean=39±6.2) and sexual abstinence was 3.8±0.8 days. Two samples were considered with inadequate cytometry pattern (outliers) and were excluded. Thus, 30 samples were divided into two aliquots so that one could have been submitted to cryopreservation/thaw and the other for lyophilization/rehydration. When we analysed ungated flow cytometry data, for cryopreserved semen samples using, death due to late apoptosis was observed in 24.2±13.9%, due to necrosis in 30.2±14.7% and initial apoptosis in 6.7±8.1%, of the studied population. Membrane integrity was preserved in 38.7±10.8% (range=14.6-59.4%). In the lyophilized samples, death due to late apoptosis was observed in 34.7±17.6%, due necrosis in 33.5±21.9% and initial apoptosis in 3.9±2.7%, of the studied population. Membrane integrity was observed in 27.6±13.5 (range=6.8-56.9%). Thus, the presence of membrane integrity was higher in the sperm population that was previously cryopreserved ($p$=0.0004). When we analysed gated flow cytometry data, for cryopreserved semen samples, death due to late apoptosis was observed in 31.1±14.5%, due to necrosis in 34.4±17.9% and initial apoptosis in 4.2±5.5%, of the studied population. Membrane integrity was preserved in 30.2±13.1% (range=12.4-68.9%). In the lyophilized samples, death due to late apoptosis was observed in 40.6±24%, due to necrosis in 42.2±26% and initial apoptosis in 5±5.4%, of the studied population. Membrane integrity was observed in 12±9.5% (range=1.4-34.3%). The presence of membrane integrity was higher in the sperm population that was previously cryopreserved ($p$=0.0001).

**Conclusion**: Lyophilization of spermatozoa is a technique with a great potential to substitute cryopreservation as there is no need for large storage space, transportation is easier, costs are lower and the risk of viral contamination is virtually eliminated as it is associated to virus inactivation. We obtained, probably for the first time, viable lyophilized human spermatozoa, with membrane integrity confirmed by flow cytometry. Although the observed percentage is still low for clinical use, we understand that lyophilization is a promising technique and that our results might encourage further studies to improve the technique until it can be routinely implemented in Assisted Reproduction clinics.

## P-05. The place reserved for man in reproduction treatments (in) fertility and (in) male visibility

**F.A.S. Giacon[1]**

Amid the isolation imposed by the danger of the new Corona virus infection, we are immersed in a "pandemic events online", to which people and professionals connect on issues of converging interests.
A question, asked during an event, on 05/30/2020, "Return to Covid Free IVF Treatments", called attention, both for its formulation and for the statistics found in the result. The question highlighted in this work was part of a quiz made at the referred event, in which the participants, mostly women who are trying to get pregnant, answered and, in the sequence, visualized the computed result. Referring to the support network during Human Reproduction treatments, she asked: "Does your husband support you on this journey?". The results obtained were: 85% of the participants answered: "Yes, a lot"; for 4% the answer was: "No, not at all" and 12% of the participants considered: "He leaves it up to me to make the decisions". This questioning and the respective collected statistics were motivating triggers for a brief bibliographic review, in the sense of understanding the place reserved for man in the Human Reproduction treatments, considering the aspects related to his (in) fertility and (in) visibility, becoming the objective of the present work. Bibliographic research methodology was chosen for a better understanding of these important aspects and to give greater visibility to man during all treatment. The justification falls on the need to foster this discussion in order to expand listening spaces for men, validating their feelings and putting them in their proper place, which is also the protagonist, co-responsible and an integral part of the treatment, and not just a supporter of the process. After the bibliographic survey, it was observed that the medical and psychological treatment generally falls on the female universe to the detriment of the male universe. As most procedures affect the woman's body, among other factors, the man is often assigned a "secondary" place in the context of Human Reproduction. At the same time, there is a growing concern, on the part of professionals, in the integration and reception of this man, who, in turn, has been taking a more proactive position in the treatment. However, it is concluded that when doubts, feelings and decisions are shared between the couple, both become stronger and create conditions to support each other, sharing the weight of the infertility trajectory, making them both protagonists throughout the treatment of Assisted Human Reproduction.

## P-06. The importance of validating new available technologies in laboratory: a study on the performance of a commercial software (Embryoscope/KIDScore™) to predict blastocyst implantation through scores subgroups evaluation.

**R.F. Erberelli[1], M. Nicolielo[1], C. Jacobs[1], F. Mendez[1], A. Belo[1], L. Cremonesi[1], I. Paranhos[1], E.L.A. Motta[2], A.R. Lorenzon[3]**

**Objective:** To validate the most recently software (KIDScore / Embryoscope™/ Vitrolife) Version 3 [V3 = 5 morphokinetics + 2 morphological parameters (TE and ICM)] comparing with Version 2 [V2 = 5 morphokinetics + 1 morphological parameters (TE)] and analyze the potential of Version 3 score to predict embryos with best implantation potential according to different score groups.
**Methods:** This is a retrospective cohort study that included patients from assisted human reproduction treatment (ART), from autologous and donated cycles using fresh and frozen oocytes, with biopsied and non-biopsied embryos transfers in a private center (Huntington Medicina Reprodutiva), between January 2018 and December 2019 using the Embryoscope® Plus incubator, that underwent single embryo transfers (sET, n=336) of blastocysts developed on day 5. Two analyzes were performed: A) KidScore ™ V2 x V3 to validate the accuracy of the system on scoring embryos that achieve a positive clinical pregnancy (presence of gestational sac and heartbeat) and negative clinical pregnancy, evaluating all patients (n = 303) and patients who only transferred euploid embryos (n = 229); B) The scores of negative and positive clinical pregnancy were separated in three groups, stratified according to V3 score intervals: Group 1, with a range between 1.0-3.9 (n = 23), Group 2, with a range between 4.0-6.9 (n = 116) and Group 3, with a range between 7.0-9.9 (n = 164). The potential of each score interval to achieve clinical pregnancy was evaluated. SET of euploid embryos (n=165) were also analyzed in the described groups (Group 1: n = 13; Group 2: n = 68 and Group 3: n = 84, respectively). Biochemical pregnancy and miscarriage were excluded. For the analysis, Paired t test, Wilcoxon, Mann-Whitney, Chi-Square and Fisher were used for statistical analysis, values of $p<0.05$ were considered significant.
**Results:** Our results showed an overall clinical pregnancy rate of 47.02% (158/336), biochemical pregnancy of 5.0% (17/336) and miscarriage of 4.76% (16/336). Maternal age between overall positive and negative pregnancies were similar (38.23±3.65 versus 38.62±3.91, $p=0.3638$, respectively). Overall, version 3 of the KIDScore™ system, when compared to Version 2, showed a greater potential to predict negative clinical pregnancy (mean score 6.33±1.87 x 6.57±1.81 $p=0.0099$). When analyzing only euploid embryos, there was no difference on V2/V3 score on negative and clinical pregnancy outcome (6.45±1.91 x 6.36±1.88, $p=0.6056$). Overall, analyzing the versions separately, the scores to predict positive and negative results showed significance, for V2 (average positive score 6.42±1.47 and negative 6.57±1.81 with $p<0.0001$) and for V3 (average positive score 7.41±1.50 and negative 6.33±1.87 with $p<0.0001$). These results were similar analyzing only euploid embryos (V2, positive 7.19±1.65 and negative 6.45±1.91, $p=0.0082$, and V3, positive 7.12±1.54 and negative 6.36±1.88, $p=0.0079$, n=87 euploid blastocysts with positive and n=78 with negative clinical pregnancy outcome). When we comparing the three score subgroups in V3, overall positive clinical pregnancy rates were significant different between the 3 groups [group 1: 17.4% (4/23); group 2: 41.4% (48/116); group 3: 64.6% (106/164) with $p=0.0001$ - Tabela I]. When analyzing group 1 versus group 2 there was also a difference in positive clinical pregnancy ($p=0.034$) and group 3 also showed a higher rate in clinical pregnancy when compared to group 1 and 2 together (scores from 1.0 to 6.9, $p<0.0001$). Analyzing only euploid embryos, the results on positive clinical pregnancy were also significant different between groups [group 1: 30.8% (4/13); group 2: 45.6% (31/68); group 3: 61.9% (52/84), $p$=0,0343 - Tabela II], and group 1 + 2 versus group 3, $p$=0,0195]. Maternal age between positive and negative clinical pregnancies after euploid sET were also similar (37.64±2.63 versus 38.32±3.42, $p$=0.1212, respectively).

**Table I.** Total

| GRUPO score TOTAL | POSITIVO (n) | NEGATIVO (n) | TOTAL (n) | Gestação Clínica (%) |
|---|---|---|---|---|
| 1 (1.0-3.9) | 4 | 19 | 23 | 17.4% |
| 2 (4.0-6.9) | 48 | 68 | 116 | 41.4% |
| 3 (7.0-9.9) | 106 | 58 | 164 | 64.6% |

Estatística (Chi-Square e Fisher)

**Table II.** Euploid

| GRUPO score EUPLOIDE | POSITIVO (n) | NEGATIVO (n) | TOTAL (n) | Gestação Clínica (%) |
|---|---|---|---|---|
| 1 (1.0-3.9) | 4 | 9 | 13 | 30.8% |
| 2 (4.0-6.9) | 31 | 37 | 68 | 45.6% |
| 3 (7.0-9.9) | 52 | 32 | 84 | 61.9% |

Estatística (Chi-Square e Fisher)

**Conclusion:** The differences on positive clinical pregnancy between subgroups of scores, according to the morphokinetic parameters, reinforce that the use of the time-lapse system in the laboratory with an in-house validated software is essential for clinical decisions, laboratory practice and understanding by patients, not only for the choice of embryos with better potential to implantation (mainly scores greater than 7.0), but also to prioritize embryos for biopsy and single embryo transfer (sET). Our results showed a slight improvement in embryo implantation prediction with the new update based on the score between version 2 and version 3 in the KIDScore™ software, mainly on embryos that will result in a negative clinical pregnancy.

## P-07. Analysis of morphokinetic parameters from blastocysts obtained from Progestin primed ovarian stimulation (PPOS) versus Antagonist protocol to inhibit LH surge. An experience of the time-lapse system in fresh egg donation cycles.

**A.P. Aquino[1], B.C. de Barros[2], L. Calazans[3], A.L.M. Campos[4], A.R. Lorenzon[5], E.L.A. da Motta[6], T.S.D. Cury[7]**

**Objective:** To evaluate and compare the morphokinetic parameters of blastocysts stage cultured in a time-lapse system obtained from fresh egg donation cycles using progestin-primed ovarian stimulation (PPOS) versus GnRH antagonist as an inhibitor of spontaneous ovulation.
**Methods:** This retrospective study aimed to compare the morphokinetic parameters from 80 blastocysts stage embryos, obtained from two different methods to inhibit LH surge on fresh oocyte donation cycles, performed between July of 2019 and March of 2020 at Huntington Group of Reproductive Medicine, Brazil (Huntington, São Paulo, and Pró-Criar, Belo Horizonte). All cycles with fresh donated oocytes were allocated into two groups according the medication used to inhibit the LH surge: group a - progesterone (PPOS protocol with dydrogesterone) and group b - standard GnRH antagonist short protocol after using hMG for ovarian stimulation. All embryos were cultured on a time-lapse system. Dydrogesterone was used 10 mg twice a day, since the first day of ovarian induction until the day of ovulation trigger, which was performed in both groups with GnRH agonist analogue (0.2 mg of triptorelin acetate) and the oocyte retrieval was done after 34-36 hours. Groups were compared on clinical aspects (basal hormone levels and antral follicle count (AFC), body mass index (BMI), length of ovarian stimulation and total dose of gonadotropin) and morphokinetic parameters on a time-lapse system (Embryoscope® Plus).
**Results:** A total of 80 blastocysts were analyzed, 46 from group a (13 patients) and 34 from group b (11 patients). All laboratory indicators parameters are described in Table 1. The following morphokinetic parameters were annotated: time of PN fading (tPNf), time to two cells (t2), three cells (t3), four cells (t4), five cells (t5), eight cells (t8), time to blastulation (tB). No statistically significant differences were observed for, tPNf: group a) 22.44±2.36h versus b) 22.92±2.93h, $p$=0.4744, t2: a) 24.89±2.40h versus b) 25.30±2.97h, $p$=0.5365; t3: a) 35.56±3.24h versus b) 35.64±3.77, $p$=0.8266; t4: a) 37.20±3.87h versus b) 36.72±4.32h, p=0,6439; t5: a) 47.09±695h versus b) 46.81±5.86h, $p$=0.7444; t8: a) 57.58±11.15h versus b) 53.16±6.78h, $p$=0.2111); tB: a) 106.42±10.94 versus b) 104.38±9.54, $p$=0.4624. Although without strong evidence on fertility potential, some differences were observed among clinical aspects (table 2), such as patients' age: a) 29.00±3.27 versus b) 25.09±3.96, $p$=0.0201, serum FSH: a) 7.71±1.25 versus b) 5.68±1.74, $p$= 0.0076, serum E2: a) 45.96±16.80 versus b) 28.09±13.67, $p$=0.0037 and total dose of gonadrotopins: a) 2123.08±474.72 versus b) 2968.18±449.04, $p$=0.0009. Statistical significances between groups were calculated using the non-parametric Mann-Whitney test.

**Table 1.** Comparison between baseline characteristics PPOS versus GnRH antagonist

| Embryonic Laboratory Indicators | PPOS (group a) n=13 patients, 46 blastocysts | GnRH antagonist (group b) n=11 patients, 34 blastocysts | p value* |
|---|---|---|---|
| No. of oocytes retrieved | 7.62±1.56 | 10±2.24 | 0.0109 |
| No. of oocytes fertilised | 6.08±1.55 | 7.55±2.11 | 0.0787 |
| No. of cleavage embryo | 5.69±1.38 | 7.09±2.07 | 0.0824 |
| No. of goog quality embryo | 4.69±1.89 | 4.73±2.37 | 0.7241 |
| No. of Blastocyst | 3.92±1.98 | 3.45±1.57 | 0.4771 |
| No. of Freeze Blastocyst | 3.92±1.98 | 3.45±1.57 | 0.4771 |
| Fertilization rate (%) | 80.57%±16.55% | 75.02%±12.56% | 0.3805 |
| Cleavage rate (%) | 95.73%±15.41% | 94.07%±9.19% | 0.142 |
| Good quality embryo rate (%) | 78.16%±27.57% | 62.91%±20.93% | 0.1065 |
| Blastocyst rate (%) | 66.18%±29.45% | 48.34%±21.83% | 0.1105 |

*Mann-Whitney test.

| **Table 2.** Comparison on clinical aspects on PPOS versus GnRH antagonist groups | | | |
|---|---|---|---|
| **Clinical characteristics** | **Group a (n=13 patients)** | **Group b (n=11 patients)** | **P value*** |
| Age | 29.00±3.27 | 25.09±3.96 | 0.0201 |
| FSH | 7.71±1.25 | 5.68±1.74 | 0.0076 |
| E2 | 45.96±16.80 | 28.09±13.67 | 0.0037 |
| BMI | 24.16±1.74 | 23.66±2.48 | 0.9538 |
| Total gonadotropin dose | 2123.08±474.72 | 2968.18±449.04 | 0.0009 |
| Days of stimulation | 10.92±1.50 | 11.45±0.82 | 0.2092 |
| AFC | 27.62±11.50 | 19.09±6.69 | 0.0584 |

*Mann-Whitney test.

**Conclusion:** The use of PPOS in ovulation stimulation protocols is getting attention as it has been demonstrated as a safe, effective and economical alternative to avoid premature LH peak. As the validation of the use of a new protocol in clinical practice should be taken into carefully consideration, here we have compared the morphokinetic parameters of the current GnRH antagonist to the PPOS protocol in egg donation cycles and no differences in these variables were found. Further analyses are needed to compare reproductive outcomes between these two protocols in our clinical practice.

## P-08. Is there any pregnancy predict factors in patients who underwent Intrauterine Insemination? 13 years of experience in a public health University Hospital

**M.R. Hentschke[1,2], V.C. Dornelles[1,2], L.M. Kobe[1], B. Cunegatto[2], D.C. Siqueira[1], V.D. Trindade[1,2], T.M. Furlanetti[1], A. Petracco[2], M. Badalotti[1,2]**

**Objective**: To analyze the profile of patients undergoing Intrauterine Insemination (IUI) and identify factors that could predict pregnancy success.

**Methods**: Observational, cross-sectional retrospective study conducted at a University Hospital (public health system), in association with a private Reproductive Medicine Center in Brazil, between 2006 to 2019. The study included 178 patients and 345 ovarian induction cycles for IUI. The indication of the procedure took place after the complete evaluation of the couple, which included the tubal and male evaluation. Participants data were analyzed from medical records and divided into two groups: group 1 (G1), patients who get pregnant (n=19) and group 2 (G2) patients who did not get pregnant (n=159). The variables age, body mass index (BMI), number of preovulatory follicles before IUI and seminal evaluation before/after preparation were compared between groups. The sperm volume preparation median was 0.3 ml. Continuous variables were presented as mean±SD or median (IIQ), and categorical variables as percentage. Student's t-test, U-Mann-Whitney of Chi-Square were applied, considering $p<0.05$.

**Results**: When analyzing the whole sample, maternal age was 34.1±4.7 and paternal age was 38.5±5.9 years old. Regarding the number of preovulatory follicle, 41.5% of all patients presented only one preovulatory follicle before IUI. The pregnancy rate was 10.6% per patient. From 19 patients who became pregnant, 11 (57.8%) were in their first follicle stimulation cycle, three (15.7%) in their second cycle, four (21%) in their third cycle and one (5.2%) in the fourth cycle. For comparative analysis between groups, only the first cycle of each patient was considered, totalizing 11 patients in G1 and 167 in G2. From the 11 patients who became pregnant, all underwent ovulation induction with clomiphene citrate associated with human menopausal gonadotropin (CC+hMG) protocol and 8 of these patients were given standard human chorionic gonadotropin (hCG) trigger. The results comparing G1 *vs.* G2 are presented in Table 1. Regarding the infertility causes, both groups presented endometriosis (72% *vs.* 53%) as the main diagnosis, followed by male infertility factor (45% *vs.* 42%) (Table 2).

| **Table 1.** Clinical and laboratory data regarding pregnant and non-pregnant patients after Intrauterine Insemination procedure. | | | |
|---|---|---|---|
| **Variables** | **G1** | **G2** | **p*** |
| Women age, years | 32.9±4.5 | 34±4.7 | 0.460 |
| Men age, years | 31±0 | 38±5.4 | 0.214 |
| BMI, kg/m² | 28.2±6 | 24.9±4.4 | 0.072 |
| Preovulatory follicles, n | 2 [1-2.25] | 2 [1-2] | 0.484 |
| Sperm volume, ml | 3 [2-4] | 2.5 [2-3.7] | 0.840 |
| Sperm Concentration, million/ml** | 35 [10-70] | 40 [15-70] | 0.659 |
| Sperm Motility,% | 50 [45-70] | 60 [50-75] | 0.356 |
| Sperm Concentration (after preparation), million/ml | 38 [7-150] | 30 [10-70] | 0.768 |

G1. Pregnant women; G2. Non-Pregnant women. BMI. Body mass index.
Variables expressed in median. *Student's t-test or U-Mann-Whitney.
**It was also performed an analysis using the cut-off point in 10 or 15 million/mL in sperm concentration before IUI and no statistical difference was observed between groups.

| **Table 2.** Infertility causes comparing pregnant and non-pregnant patients after Intrauterine Insemination procedure. | | | |
|---|---|---|---|
| **Variables** | **G1** | **G2** | **p*** |
| PCOS | 1/11 (9%) | 27/166 (16%) | 0.528 |
| Endometriosis | 8/11 (72%) | 88/166 (53%) | 0.204 |
| Male factor | 5/11 (45%) | 70/166 (42%) | 0.831 |
| Tubal Factor | 0/11 (0%) | 18/166 (10%) | - |
| Non-cause | 1/11 (9%) | 27/166 (16.2%) | 0.528 |

G1. Pregnant women; G2. Non-Pregnant women. PCOS. Polycystic Ovary syndrome.
Variables expressed in percentage. *Chi-square test

**Conclusion**: In the present study, there was no predict factors found in patients who underwent IUI. However, it was observed the presence of tubal factor only in the group of patients who did not become pregnant. Also, the sample size and its heterogeneity may justify the lack of significance in the results. It is important to emphasize that, in some situations, the patient would have more benefit from *in vitro* fertilization (IVF); however, as public health system users have difficult access to this procedure, many of them performs IUI even knowing its limited chances, which seems to be still higher than spontaneous pregnancy. The fact of having both groups with the same characteristics leads us to think of the real benefit of the procedure *per se*. Moreover, would be grateful having a control group with patients who get pregnant spontaneously or after ovulation induction to better analyze the results.

## P-09. Morphokinetic Analysis of Blastocysts from frozen and fresh oocytes in Progestin Primed Ovarian Stimulation (PPOS) Protocol

**L.C. Calazans[1], A.L.M. Campos[2], C.C. Moraes[2], B.C. Barros[3], A.P. Aquino[4], A.R. Lorenzon[5], T.S. Domingues[6], J.P.J. Caetano[7]**

**Objective:** To compare the laboratory and morphokinetic indicators of embryos from egg donation cycles with fresh and frozen oocytes, in cycles with Progestin Primed Ovarian Stimulation (PPOS) Protocol, which uses progestins for pituitary blockage of premature LH peak during ovarian stimulation.
**Methods:** A retrospective study included the egg donation cycles performed at Pró-Criar Reproductive Medicine Center, Belo Horizonte, MG, Brazil, between July 2019 and March 2020 using PPOS protocol to block the LH peak during ovarian stimulation of the egg donors. Ovarian stimulation started after ultrasound on the 2nd-3rd day of the spontaneous menstrual cycle or after the 4th-5th day of pause of the combined oral contraceptive to evaluate the pituitary blockade, with hMG used at ranges from 225-300 IU daily. The initial and continuous gonadotropin dosages were adjusted according to patient age, baseline FSH level, body mass index (BMI), antral follicle count (AFC) and response to follicular growth. Dydrogesterone 10 mg twice daily was started on the first day of ovarian induction and maintained until the day of ovulation trigger, which was performed with GnRH agonist analogue (0.2 mg of triptorelin acetate). Ovarian puncture was performed 34-36 hours after the trigger injection. All cycles with donated oocytes, fresh or frozen, from donor cycles submitted to PPOS were included. All embryo development cycles evaluated were cultured in the incubator with EmbryoScope Plus® time-lapse technology. The rates of fertilization, cleavage and blastocyst formation were evaluated, in addition to the embryonic morphokinetic parameters: time of appearance of the pronuclei (tPNf), time for cleavage in two cells (t2), time for cleavage in three cells (t3), time for cleavage in four cells (t4), time for cleavage in five cells (t5), time for cleavage in eight cells (t8) and the time for the first blastulation signal (tB).
**Results:** A total of 22 cycles and 82 blastocysts were analyzed. 13 cycles and 46 blastocysts were from recipients that received fresh oocytes and 9 cycles and 36 blastocysts were from recipients that received frozen oocytes. The baseline characteristics of fresh and frozen egg donors were similar, and there was no difference at the number of oocytes collected [Age mean 29.00±3.27 X 29.56±3.09 (*p*=0.7368) years old, BMI mean 24.16±1.74 x 23.68±2.58 (*p*=0.7125) Kg/m², basal FSH mean 7.71±1.25 x 7.16±1.45mUI/mL (*p*=0.2689), AFC mean 27.62±11.50 x 23.89±11.94 (*p*=0.3829) and MII mean 15.0±7.98 X 15.89±8.43 (*p*=1.0000)]. The recipients of the fresh group received 7.62±1.56 oocytes, and those of the frozen group 7.56±1.33, in average, without significant difference (*p*=0.917). The fertilization rate was similar between the groups (80.57% x 69.10%, *p*=0.105), as well as the cleavage rates (95.73% x 95.00%, *p*=0.4336) and formation of blastocysts (66.18% x 55.77%, *p*= 0.365). There were also no significant differences in the morphokinetic parameters of the embryos evaluated: tPNf was 22.44±2.36hours in the fresh group and 23.55±3.78hours in the frozen group (*p*=0.168). t2 24.89 x 26.05hours (*p*=0.220), t3 35.56 x 37.20 hours (*p*=0.337), t4 37.20 x 39.71hours (*p*=0.246), t5 47.09 x 48.51hours (*p*=0.736), t8 57.58 x 59.63hours (*p* = 0454) and tB 106.42 x 109.52 hours (*p*=0.705).
**Conclusion:** No significant differences were observed in the laboratory and morphokinetic parameters analyzed. For our egg recipients, when eggs where obtained with PPOS protocol, there was no difference between using fresh versus frozen eggs, in regard of the embryonic outcomes.

## P-10. Dydrogesterone as an alternative to suppress LH surge in ART cycles

**M.C.B. Souza[1], A.C.A. Mancebo[1], M.M. Souza[1], R.A. Antunes[1], L.B.A. Silva[1], A.L.O. Barbeitas[1], V.A. Raupp[1]**

**Objective**: Progesterone used since the beginning of follicular phase (progestin primed ovarian stimulation - PPOS) has been recently described concomitant with exogenous gonadotrophin in ART cycles. They point to an useful strategy, greater practicality and lower cost. The aim of this study is to evaluate the effectiveness of Dydrogesterone (DYG) in PPOS protocols for IVF/ICSI cycles and Oocyte cryopreservation. Objective: To compare the use of either GnRH antagonist or dydrogesterone to suppress LH premature surge during ovarian stimulation in IVF/ICSI cycles or Oocyte Cryopresevation .
**Methods**: 65 IVF/ICSI plus 15 oocyte cryopreservation cycles from October 2018 to February 2019, without age restriction. Follitropin delta (Rekovelle®, Ferring Pharmaceuticals) were given in a fixed daily SC dose determined by serum AMH level assessed by the automated Elecsys AMH immunoassey® Roche (ng/mL) and body weigth (kg), according to the Rekovelle algorithm. Clinical decision led to a GnRH antagonist (CTA-Cetrorelix acetate, Cetrotide®, Merck) 0.25mg/d initiated in a flexible shedule in presence of one follicle ≥14mm and continued throughout the stimulation period (35 cycles) or dydrogesterone (DYG), 10mg 8/8hs (Duphaston®, Abbott) combined to follitropin delta from the beginning of stimulation until the day after the trigger (45 cycles). The final follicular maturation was performed when there were three or more follicles ≥17mm diameter either with 250mg recombinant hCG (Choriogonadotropin alfa, Ovidrel®, Merck) or GnRH agonist, 2 ampules (triptorelin acetate Gonapeptyl Daily®, Ferring Pharmaceuticals). Criteria for cancelling: clinician judgement if no follicles with a diameter 17 mm by day 15. Oocyte retrieval took place 36 hours after trigging. Primary outcome was the incidence of premature LH surge. Secondary outcomes included follicles ≥16 mm and ≤19mm and ≥20mm on hCG day, metaphase II oocytes, the cancelling of cycles and OHSS symptoms. Statistics were performed by Mann-Whitney test.
Results: DYG group had a mean age significantly higher (36.4 x 38.26-p 0.01). There were no differences in mean parameters of BMI (25.3 x 23.4), days of stimulation (9.2 x 9.3), AMH values (2.3 x 2.3), AFC (15.0 x 15.2). In the same way, no differences were observed between follicles 16 mm to 19 mm (3.6 in both groups), ≥ 20mm (2.2 x 3.0), metaphase II oocytes (6.7 x 7.5), metaphase I (0.5 x 0.3) and GV (1.8 x 1.6). No patient from either group experienced a premature LH surge (mean LH at the trigger day 3.0 x 3.6, not significant) and one case in the DYG group had no oocyte in a single 20 ml aspirated follicle. There happened no cancelling. 59% of DYG patients used 12 mcg/day of Rekovelle, as well as 47% of the CTA group. No patients experienced moderate to severe ovarian hyperestimulation syndrome, even when AMH > 3 ng/mL.

Data expressed as averages ±SD

**Conclusions:** Dydrogesterone is an eligible tool to IVF/ ICSI cycles intended to freeze-all / PGT-A and oocyte preservation. It is to be considered either to embryo banking or preventing OHSS in higher AMH patients, added in follitropin delta protocol.

## P-11. Clinical pregnancy in blastocyst transfer is correlated to blastulation morphokinetic parameter

**C. Jacobs[1], M. Nicolielo[1], F. Mendez[1], R. Eberelli[1], A. Belo[1], D. Reis[1], C. Fauth[1], E. L. A. Motta[2], AR. Lorenzon [3]**

**Objective:** To evaluate the morphological and morphokinetic parameters of blastocyst stage embryos with known reproductive outcome from an oocyte donation program.

**Methods:** Blastocysts with known reproductive outcome after single embryo transfer (sET) or double embryo transfer (dET) from patients undergoing assisted reproduction treatment in the oocyte donation program between October 2017 and December 2019 were included in this study. Biopsied blastocysts were excluded from this study. Embryos were cultured in a time-lapse system incubator and had the following morphokinetic parameters annotated in hours (h): time of pronucleous fading (tPNf), time to 2-cell (t2), time to 3-cell (t3), time to 4-cell (t4), time to 5-cell (t5), time to 8-cell (t8) and time to blastulation (tB). Embryos were morphologically graded according to Gardner and Schoolcraft criteria.

**Results:** One-hundred forty-nine embryos with known reproductive outcome were analyzed. Ninety blastocysts resulted in positive clinical pregnancy (63.8%, 58 from sET and 32 from dET) and 59 in negative (36.2%, 22 from sET and 37 from dET) after 133 fresh embryo transfers and 14 frozen embryo transfers in cycles with thawed oocytes and 2 frozen embryo transfers following fresh oocytes fertilization. Oocyte donors average age is 24.68±3.89 years. The analyzed parameters are described in table 1. The time of blastulation (tB) morphokinetic parameter was significantly earlier in embryos that achieved clinical pregnancy (tB: 102.52±6.72 h versus 106.37±7.90h *p*=0.0005). Regarding other parameters analyzed, all earlier time-points were similar between positive and negative clinical pregnancy (tPNf: 22.98±2.80h versus 23.52±2.66h, *p*=0.1591, t2: 25.77±3.01h versus 2622±2.84h, *p*=0,3213; t3: 36.77±3.61h versus 36.76±3.54h, *p*=0.8781; t4: 37.90±4.29h versus 38.17±4.72h, *p*=0.9034; t5: 49.79±5.84h versus 48.54±6.76h, *p*=0.4139; t8: 56.93±8.64h versus 58.46±9.00h, *p*=0.2476). Morphology grade for positive and negative clinical pregnancy were different between good quality embryos (grades A and B – 95,6% versus 76.3%, respectively) and poor quality embryos (at least one grade C – 4.4% versus 27.7%, respectively *p*=0.0006). Statistical significances were calculated using Fisher or t-test as appropriate.

**Table 1.** Comparison between positive and negative clinical pregnancy and embryos morphology and morphokinetic parameters.

| Variable | Positive clinical pregnancy (n=74, 63.8%) | Negative clinical pregnancy (n=42, 36.2%) | *p*-value |
|---|---|---|---|
| TPNf | 22.98 ±2.80 h | 23.52 ±2.66 h | 0.1591 |
| t2 | 25.77 ±3.01 h | 26.22 ±2.84 h | 0.3213 |
| t3 | 36.77 ±3.61 h | 36.76 ±3.54 h | 0.8781 |
| t4 | 37.90 ±4.29 h | 38.17 ±4.72 h | 0.9034 |
| t5 | 49.79 ±5.84 h | 48.54 ±6.76 h | 0.4139 |
| t8 | 56.93 ±8.64 h | 58.46 ±9.00 h | 0.2476 |
| tB | 102.52 ±6.72 h | 106.37 ±7.90 h | 0.0005 |
| Good morphology (A and B grades) | 95.6% | 76.3% | #0.0006 |
| Poor morphology (at least one C grade) | 4.4% | 23.7% | |

Mann-Whitney t-Test / #Fisher test

**Conclusion:** Our results highlight the earlier time to blastulation in the embryo group that achieved clinical pregnancy. This morphokinetic parameter also correlates to better morphology blastocysts, consequently leading to higher success rates. Since the embryos analyzed were generated from an oocyte donation program, the maternal age impact in oocyte quality was excluded. The difference observed in time to blastulation represent a potential time-point to be better analyzed in laboratory algorithms, and reinforces the potential use of morphokinetics as a tool for non-invasive embryo selection.

## P-12. Graphic projection analysis as a psychological diagnosis tool in new family constitutions undergoing assisted reproduction treatment

**R.M.M. Melamed[1], D.P.A.F. Braga[1,2], Edson Borges Jr.[1,2]**

**Objective:** There are some themes that cause controversy concerning the advances in assisted reproduction techniques and the constitution of new family nuclei. The search for assisted reproduction treatments, by new family constitutions, to achieve parenthood, with biological children has increased considerably. The goal for the present study was to, by using graphic projection techniques, investigate the emotional response and possible changes in patients with poor or absent ovarian reserve ovarian reserve and therefore presenting an obstacle to procreation, and also the emotional issues associated with the uterus surrogacy.
**Methods**: The investigation was divided into three phases: (i) the interview, (ii) drawing of the human figure and family, and (iii) the inquiry. Six patients participating in two different situations were included in the study. SITUATION A: a heterosexual couple, both over 45 years old. The woman was diagnosed with ovarian failure and uterine malformation, therefore, the pregnancy was discouraged due to a negative prognosis. The couple was counselled to undergo uterus surrogacy, being the surrogate mother a first-degree relative. SITUATION B: a male homosexual couple, the oldest being over 35 year old and the youngest with 28 years old. To have your own baby the couple will undergo anonymous ovum donation, and the eggs inseminated by the older men sperm. In addition, the embryo will be transferred to a first-degree relative to the youngest men, as a surrogate mother.
**Results:** We observed that the three participants who would not use their own oocytes, two male and one female, drew an incomplete human figure, presenting only the face. When the family drawing was requested, its elements appear complete, with body and face. A similar result was observed in a previous study of our group, in which two patients with non-obstructive azoospermia also drew incomplete human figures, presenting exclusively the face, and again when asked to draw the family, the human figure appeared complete. These patients underwent unsuccessful surgical sperm retrieval and used the sperm banking to have their own child. When the surrogate mothers were asked to draw human figures, the human figure was complete and the number of family members corresponded to the number of people of their current nucleus.
**Conclusion:** In conclusion, drawing is an extremely useful investigation tool, which in addition to projecting the body image; it composes a range of projections, when associated with psychological interviews. Therefore, this approach provides elements for the psychological treatment in patients undergoing assisted human reproduction and, in some cases, helping to deal with the absence of its own genetics on their future descendants

## P-13. Morphokinetic Analysis of Blastocysts obtained after Progestin Primed Ovarian Stimulation (PPOS) and Double Stimulation (DUOSTIM)

**ALM Campos[1], LC Calazans[1], LMR Pereira[1], LMA Maia[1], LVC Amorim[1], BCL Guimarães[1], RM Marinho[1], JPJ Caetano[1]**

[1]*Huntington/Pró-criar Medicina Reprodutiva*

**Objective:** To compare the morphokinetic indicators of the blastocysts obtained after folicular phase and luteal phase stimulations (FPS and LPS) in the same ovarian cycle (DuoStim) with Progestin Primed Ovarian Stimulation (PPOS) Protocol.
**Methods:** A retrospective case-control study compared the morphokinetic indicators of the blastocysts obtained from FPS and LPS, in the same patientes. It included 37 blastocysts from 8 patients undergoing DuoStim for In Vitro Fertilization (IVF) /Intra-cytoplasmic sperm injection (ICSI) at Pró-Criar Reproductive Medicine Center, Belo Horizonte, Minas Gerais, Brazil between march 2019 and march 2020. FPS and LPS were performed with the same daily dose of gonadotropins and with PPOS Protocol.
The FPS started after ultrasound on the 2nd-3rd day of the spontaneous menstrual cycle or after the 4th-5th day of pause of the combined oral contraceptive to evaluate the pituitary blockade (endometrium smaller than 5mm and suppressed ovaries with absence of follicles larger than 10 mm). The type of gonadotropin used did not follow a pattern as it was left to the discretion of the attending physician; both recombinant FSH and hMG were used at ranges from 150-300IU daily. The initial and continuous gonadotropin dosages were adjusted according to patient age, baseline FSH level, body mass index (BMI), antral follicle count (AFC) and response to follicular growth. Dydrogesterone 10 mg 12-12 hour started on the first day of ovarian induction and was maintained until the day of ovulation trigger, with GnRH agonist analogue (0.2 mg of triptorelin acetate). Ovarian puncture was performed 34-36 hours after the trigger injection. After 5 days of from the first retrieval, LPS was started with the same protocol and same daily dose of gonadotropins as the LPS, without ultrasound monitoring. Gonadotropin dosages adjustments were conducted the same way as in the FPS and GnRH agonist analogue (0.2 mg of triptorelin acetate) was used for ovulation trigger. All embryo development cycles evaluated were cultured in the incubator with EmbryoScope Plus® time-lapse technology. The rates of fertilization, cleavage and blastocyst formation were evaluated, in addition to the embryonic morphokinetic parameters: time of appearance of the pronuclei (tPNf), time for cleavage in two cells (t2), time for cleavage in three cells (t3), time for cleavage in four cells (t4), time for cleavage in five cells (t5), time for cleavage in eight cells (t8) and the time for blastulation (tB).
**Results:** A total of 37 blastocysts from 16 cycles (8 FPS and 8 LPS cycles) were analyzed. 18 blastocysts were obtained from the FPS and 19 blastocysts from the LPS. The 8 patients analysed were their own control, and had mean age of 3663 (± 4.57 ) years old, mean BMI of 22.79 (± 2.50) and mean antral folicular count of 10.14 (± 2.73). There were no significant differences in the morphokinetic parameters evaluated: tPNf was 23.12±2.24 in the FPS group and 23.01±2.33 in LPS group ($p$=0.883); t2 25.52±2.30 x 25.62±2.45 ($p$=0.906); t3

36.55±2.92x36.70±4.67 ($p$=0.910); t4 37.97±4.03 x 38.08±3.08 ($p$=0.929); t5 49.49±4.64 x 51.04±5.16 ($p$=0.950); t8 57.34±5.90 x 57.47±6.46 ($p$=0.950) and tB 106.20±12.48x108.90±11.24 ($p$ =0.499).
**Conclusion:** No significant differences were observed in the morphokinetic parameters analyzed between FPS and LPS blastocysts of the same ovarian cycle, when PPOS protocol was used in both stimulations. DuoStim is a good option to maximize the exploitation of ovarian reserve in the same menstrual cycle in comparison to conventional stimulation, without worsening the morphokinetic parameters of the blastocysts produced in the second stimulation.

## P-14. Stress and anxiety of women submitted to assisted human reproduction

**B.R. De Marchi[1], L.F. Previato[1], C.L. Fácio[1], L.A. Machado-Paula[1], E. Araujo Filho[1]**

**Objective:** The aim of the study was to investigate and compare the levels of stress and anxiety in women undergoing Assisted Human Reproduction Treatment (TRHA) before and after in vitro fertilization (IVF); as well as raising sociodemographic and clinical characteristics; verify correlation of stress and anxiety in maturity and oocyte quality and in the final result; compare stress and anxiety before and after the procedure.
**Methods** It is a descriptive, longitudinal research. The participants answered the Sociodemographic Questionnaire, the Beck Anxiety Inventory (BAI) and the Lipp Stress Symptoms Inventory (ISSL).
**Results:** 26 women participated in the study. The mean age was 31.5 years (± 2.54); married, with an average of 3.5 years of natural pregnancy attempts (± 2.57). Of the sample, fourteen (53.84%) had a female factor as impeding factor, six (23.07%) male factor, three (11.53%) female factor plus male factor, and three (11.53%) without cause apparent. The results of BAI indicated that twenty (76.93%) did not show symptoms of pre-treatment anxiety, three (11.53%) mild anxiety, one (3.85%) moderate and two (7.69%) severe. In the post-treatment period, twenty (76.93%) had no anxiety and six (23.07%) were mild. Regarding anxiety, there was no correlation between pre- and post-treatment symptoms with oocyte quality and final result (Beta HCG). The ISSL results indicated that twenty (76.93%) presented stress in the pre-treatment alert phase and six (23.07%) in the resistance phase. In the post-treatment, twenty-one (80.77%) presented stress in the alert phase and five (19.23%) presented in the resistance phase. It was possible to identify that twenty of the participants (76.93%) had no symptoms of stress in the pre and post treatment and with that, there was no significant correlation in the oocyte quality ($p$=0.0027) in the MII stage, however in relation to the Beta There was no correlation with HCG symptoms of stress. Regarding the laboratory report, the median of captured oocytes was 15 (range 3 to 41); mature oocytes (MII) of 11 (range 3 to 28); immature oocytes (MI) 0.9 (range 0 to 5); immature oocytes (PI) of 0.7 (range 0 to 5), and ruptured oocytes of 0.6 (range 0 to 6). Beta HCG was seventeen (65.38%) positive and nine (34.61%) negative.
**Conclusion:** The study participants did not show significant symptoms of stress and anxiety. Psychological monitoring helped to avoid high levels of stress and anxiety, but further research is needed in the area to show the effectiveness of psychological monitoring in TRHA and the importance of this role with multidisciplinary Assisted Reproduction teams.

## P-15. Pregnancy rate in relation to embryo quality of fresh embryos in D2/D3 versus D5.

**A. Castelo Branco[1], A.C.L. Fontes[1], S. Nachef[1]**
[1]*Art Fértil Clínica de Reprodução Humana, Recife, PE, Brasil.*

**Objective**: To compare the pregnancy rate in relation to the embryo quality of fresh embryos in D2/D3 versus D5.
**Methods**: Retrospective study, performed at the ART FÉRTIL clinic, Recife, PE. Between January 2018 and March 2020, 351 patients underwent IVF treatment with intracytoplasmic sperm injection, with fresh transfers. The mean age of the patients was 36 years (25-46 years). The embryo quality was divided into: group I (good quality), group II (median quality) and group III (poor quality).
**Results**: In fresh transfers, the pregnancy rate in group I was 35.75% (n=59) in D2/D3 embryos (n=165) and 57.40% (n=62) in D5 embryos (n=108). In group II, the pregnancy rate was 25.92% (n=07) in D2/D3 embryos (n=27) and 41.66% (n=05) in D5 embryos (n=12). And in group III, it was 11.42% (n=04) in D2/D3 embryos (n=35) and 0% (n=0) in D5 embryos (n=4).
**Conclusion**: Transfer in D5 may be prioritized in cases where the patient has 5 or more D2/D3 embryos in groups I and II, with the aim of selecting at least 01 blastocyst. However, in cases where the patient has few D2/D3 embryos (up to 03) in groups I, II and III, and consent to transfer 02 or 03 embryos depending on the patient's age, the transfer can be performed at this stage, in order to avoid further exposure of embryos and manipulation in the laboratory. This shows us the importance of individualizing each patient, and what is the most appropriate time for embryonic transfer.

## P-16. Seminal fluid parameters and varicocele effects: the reality of a public service.

**U.B. Silva[1], J.R.I. Lopes[1], L.C.A. Silva[1], M.M.G. Torres[1], A.L.B. Santanna[1], M.Q.L.M. Martins[1], E.J.P.B. Nóbrega[1], K.R.N. Barbosa[1], J.F. Aderaldo[1]**

**Objective**: Infertility is perhaps the best studied, most complex, and enigmatically still one of the most controversial aspects of varicoceles and urology in general. From a population perspective, 16% of men with confirmed fertility had a varicocele at the time of vasectomy. There is still an ongoing debate among researchers as to if and to what extend varicocele affects semen parameters, which usually vary from normal to mild or moderate asthenospermia, teratospermia or asthenoteratospermia. It seems that fertile and infertile men with varicocele have similar semen parameters with those without the condition. Thus, it is speculated that varicocele affects fertility and sperm quality in some, but not in all men. Another assumption is that sperm quality is not affected by varicocele as such, but simply coexists in some men with idiopathic infertility and abnormal semen parameters. Others have shown that most men with a varicocele have normal semen parameters, suggesting a complex interplay between infertility and the presence of a varicocele. The present study aims understand how the semen parameters are affected by the varicocele condition and show if it affect the male fertility.

**Methods**: A retrospective chart review was performed with fifty one patients between 14 and 48 years of age, and all then present varicocele. The patients had semen analyses carried out in the same public andrology laboratory in Brazil from 2016 to 2019. The three most critical seminal parameters (concentration, motility and morphology) were compared acoording WHO (world health organization) parameters. The parameters established by the WHO for the categories were considered: Oligospermia (sperm count ≤15 million/ml); Severe Oligospermia (sperm count ≤5 million/ml); Atenozoospermia (total motile sperm < 40%); and Teratozoospermia (normal forms <4%, according to the Kruger criterion). For the statistic analise, the Shapiro-Wilk normality test was applied to verify the adherence of continuous variables to the normal distribution. The Student's t test for paired samples was applied to continuous variables that showed normality and the Wilcoxon test was applied to those that did not show normality. Fisher's exact test was used to analyze the association between categorical variables. The significance level of 5% was adopted for all analyzes.

**Results:** From the analysis of the data, it can be seen that patients affected by varicocele suffer some reduction in fertility with regard to all analysis parameters. More than fifty percent of pacients showed a great reduction on sperm concentration and morphology and twenty percent present spermatozoids motility affected.

**Conclusion**: A large scale study by the WHO showed significantly lower sperm concentration in infertile men with varicocele, compared to men with idiopathic infertility, but did not give any evidence regarding motility and morphology of the sperm. Although, according a recent review about the topic, varicocele clinically detected is a significant risk factor for decreased sperm count, motility, and morphology in adult infertile men with confirm our analysis. However, others studies says: semen does not seem to be affected for varicocele, because they revealed that infertile men and men of the general population with or without varicocele do not present any significant difference regarding the semen parameters. So, this shows the necessity of a better understanding of the collective influence of varicocele on sperm quality and subsequently fertility will help improve treatment, and support for affected individuals.

## P-17. Progestin Primed Ovarian Stimulation (PPOS) Protocol plus Double Stimulation (DUOSTIM) to inhibit LH peak: a plausible strategy for IVF cycles.

**L.M.A. Maia[1], J.P.J. Caetano[1], L.C. Calazans[1], L.M.R. Pereira[1], L.V.C. Amorim[1], A.L.M. Campos[1], C.C. Moraes[1], R.M. Marinho[1]**

[1]*Huntington/Pró-criar Medicina Reprodutiva*

**Objective:** To compare the mean number of oocytes and blastocyst formation rate after folicular phase and luteal phase stimulations (FPS and LPS) in the same ovarian cycle (DuoStim) with Progestin Primed Ovarian Stimulation (PPOS) Protocol.

**Methods:** A retrospective case-control study compared the results of FPS and LPS in the same patients. It included 13 patients undergoing DuoStim for In Vitro Fertilization (IVF) / Intra-cytoplasmic sperm injection (ICSI) at Pró-Criar Reproductive Medicine Center, Belo Horizonte, Minas Gerais, Brazil, between march 2019 and march 2020. FPS and LPS were performed with the same daily dose of gonadotropins and with PPOS Protocol. The FPS started after ultrasound on the 2nd-3rd day of the spontaneous menstrual cycle or after the 4th-5th day of pause of the combined oral contraceptive to evaluate the pituitary blockade (endometrium smaller than 5mm and suppressed ovaries with absence of follicles larger than 10 mm). The type of gonadotropin used did not follow a pattern as it was left to the discretion of the attending physician; both recombinant FSH and hMG were used at ranges from 150-300 IU daily. The initial and continuous gonadotropin dosages were adjusted according to patient age, baseline FSH level, body mass index (BMI), antral follicle count (AFC) and response to follicular growth. Dydrogesterone 10 mg 12-12 hour started on the first day of ovarian induction and was maintained until the day of ovulation trigger, with GnRH agonist analogue (0.2 mg of triptorelin acetate). Ovarian puncture was performed 34-36 hours after the trigger injection. After 5 days of from the first retrieval, LPS was started with the same protocol and same daily dose of gonadotropins as the LPS, without ultrasound monitoring. Gonadotropin dosages adjustments were and conducted the same way as in the FPS just like the trigger (0.2 mg of triptorelin acetate). The mean number of antral follicules, oocytes, mean number of top quality cleavage stage embryos and mean number of blastocysts were calculated for the FPS and LPS.

**Results:** The study was carried out with 13 women with a mean age of 38.54 years (SD 4.43 years) and an average BMI of 25.71 kg/m$^2$ (SD 5.92kg/m$^2$). And the mean number of antral follicles was 9.25 (SD 3.19). In comparison between cycles on DuoStim, FSP was 2 day shorter than LPS (10,31 ± 1.93 vs. 12.46±3.02; $p$=0,096) and the total dose of gonadotropin in FSP was smaller than LPS (2.187IU±475.18 vs 2.738IU±631.96; $p$=0.096), nevertheless was no significantly difference on two groups. Overall, 60 and 70 MII oocytes were retrieved after FPS and LPS, respectively; 24 blastocysts formed on FPS cycle and 27 blastocysts formed on LPS in the ovarian cycle. No difference on data analysis between the two groups were significantly correlated. On average, mature oocytes (MII) collected after FPS and LPS was (4.62±2.02 vs. 5.39±2.93; $p$=0.316). The mean on embryos formed was similar among the two paired groups. The average number on fertilized embryos was 3.77±1.74 in the FPS group and 4.46±2.30 in LPS group ($p$=0,289); the embryos on 3rd day cleavage was 3.15±1.52 in the FPS group and 3.54±1.85 in LPS group ($p$=0.550); and the number of blastocysts obtained 1.85±0.90 in the FPS group and 2.08±1.66 in LPS group ($p$=0,649).

**Conclusion:** Interestingly, both number of mature oocytes and the number of blastocysts obtained did not show any difference statistic among FPS and LPS, suggesting that the two cohorts of oocytes retrieved are equivalent from a global analysis, but not evaluated from an intrapatient analysis of each DuoStim cycle. The DuoStim associated with Progestin Primed Ovarian Stimulation is a plausible protocol and could be a strategic about time for treatment with the intent to increase the number of oocytes retrieved and the blastocysts available for transfer or for PGT-A. This approach could be a good choice for reduce treatment time and patient drop-out.

## P-18. Unveiling the surrogacy: the relationship between the baby´s biological parents and the "solidary belly" woman during and after pregnancy

**S.L. Silva, R. C. S.Lopes**

*No institutional affiliation*

This work aims to address and provide reflections on the relationship established between the couple, the baby's biological parents, and the "solidary bellies" women who chose to carry out the surrogacy. It is a retrospective, qualitative and exploratory research. Therefore, was carried out a study of multiple cases involving three women who agreed to lend their wombs to gestate the embryo of a heterosexual couple without receiving any payment for the pregnancy. The "solidary bellies" women were interviewed by videoconference and responded to a semi-structured interview about the experience of surrogacy. All interviews were transcribed, analyzed and each case was described through a clinical report that made it possible to observe the uniqueness of the experience of surrogacy, as well as the relationship between the "solidary belly" woman and the couple for whom she gestated. It is worth mentioning that it was decided to name the participants in this way, instead of a surrogate mother, because they called themselves "solidary belly" during data collection. It was observed that the experiences were permeated by ties of familiarity in one case, and friendship in the other two cases. The relationship between women "solidary bellies" and the biological mothers of babies already existed before the replacement pregnancy proposal occurred and was one of the factors that motivated "solidary bellies" women to accept gestating babies. Besides, during pregnancy, the relationship between them became closer and continues until nowadays, as all participants became godmothers of the babies they gestated. Regarding the relationship between the "solidary bellies" women and the babies' biological fathers, it was found that there was an approach of affection marked by the family bond and frequent interaction with the pregnant woman in one of the cases.

In another case, the father showed himself to be attentive and concerned with the well-being of the pregnant woman, but without establishing any more intimate emotional bonds while respecting the bonds of friendship between them. In the other case, the father remained distant from the "solidary belly" woman, having little participation in the substitution pregnancy experience. It is concluded that the relationship of the "solidary bellies" women with the baby's mothers has become increasingly intimate and the bond established between them remained after delivery and the delivery of the baby. However, the relationship with the fathers of the babies occurred differently in the three cases, as the relationship established between them during pregnancy was influenced by the bonds established before her. It is noteworthy that the possibility of speech provided by the research made it possible for them to put veiled experiences into words in their unique paths of surrogacy. Also, it is hoped that this study promotes visibility to the phenomenon, provides reflections, and contributes to the strengthening of public policies that ensure care for the psychic health of the surrogate mother, as well as for all those involved.

## P-19. Double Stimulation (DUOSTIM) for elective oocyte freezing: are follicular phase stimulation and luteal phase stimulation comparable?

**L.V.C. Amorim[1,2], L.M.R. Pereira[1], L.C. Calazans[1], LMA Maia[1], E.B.S. Xavier[1], A.L.M. Campos[1], T.H.S. Olintho[1], R. Marinho[1], J.P.J. Caetano[1]**

*[1]Huntington/Pró-criar Medicina Reprodutiva*

**Objective:** To compare the number of metaphase II (MII) oocytes obtained after follicular phase and luteal phase stimulations (FPS and LPS) in the same ovarian cycle (DuoStim).

**Methods:** A retrospective case-control study compared the results of the number of MII retrieved in FPS and LPS at the same patients. It included 19 patients undergoing DuoStim for Elective Oocyte Freezing at Pró-Criar Reproductive Medicine Center, Belo Horizonte, Minas Gerais, Brazil between march 2019 and march 2020. FPS and LPS were performed with Progestin Primed Ovarian Stimulation (PPOS) and/or Antagonist Protocol. The protocol and the dose of gonadotropins used varied according to assistant physician. The FPS started after ultrasound on the 2nd-3rd day of the spontaneous menstrual cycle or after the 4th-5th day of pause of the combined oral contraceptive to evaluate the pituitary blockade (endometrium smaller than 5mm and suppressed ovaries with absence of follicles larger than 10 mm). The type of gonadotropin used did not follow a pattern as it was left to the discretion of the assistant physician; both recombinant FSH and hMG were used at ranges from 150-300 IU daily. The initial and continuous gonadotropin dosages were adjusted according to patient age, baseline FSH level, body mass index (BMI), antral follicle count (AFC) and response to follicular growth. GnRH agonist (0.2 mg of triptorelin acetate) or human chorionic gonadotropin (hCG) were used as ovulation trigger. Oocyte pick-up was performed 34-36 hours after the trigger injection. After 5 days of from the first retrieval, all patients in LPS was started with PPOS protocol and same daily dose of gonadotropins as the FPS, without ultrasound monitoring. Gonadotropin dosages adjustments were conducted the same way as in the FPS.

**Results:** Of the 19 patients analyzed, 8 used Antagonist Protocol in the FPS, and PPOS in the LPS. The other 11 patients used PPOS in both stimulations. In the first stimulation the trigger was performed with GnRH agonist in 18 patients. For 1 patient the trigger was hCG. In the second stimulation 13 used hCG and 6 used GnRH agonist. The mean age was 36.8 years (SD 2.3 years), the average BMI was 23.1 kg/m$^2$ (SD 2.52 kg/m$^2$) and the mean number of antral follicles was 12.9 (SD 6.2). The mean number of MII obtained was 5 in FPS and 6 in LPS ($p$=0.647). Duration of ovarian stimulation was 11 days in FPS and 12.5 days in LPS ($p$=0.015). The average dose of gonadotropin used was 2,380 IU in FPS and 2,791 IU in LPS ($p$=0.026).

**Conclusion:** In this study, there was no difference between the outcomes in relation to the average number of MII obtained. LPS showed similar competence in number of oocyte retrieved at FPS. However, for LPS the mean stimulation time was longer and the dose of gonadotropin used was higher. This protocol could be a good choice to reduce the patient dropout and to increase the total number of frozen oocytes.

## P-20. Effect of varicocelectomy on sperm quality in a public Andrology service in Brazil.

**J.R.I. Lopes[1], L.C.A. Silva[1], U.B. Silva[1], M.M.G. Torres Torres[1], A.L.B. Santanna[1], M.Q.L.M. Martins[1], E.J.P.B. Nóbrega[1], K.R.N. Barbosa[1], J.F. Aderaldo[1]**

**Objective**: To observe the impact of varicocelectomy on the most critical seminal parameters.
**Methods**: A retrospective chart review was performed with twenty patients between 24 and 48 years of age. The patients had semen analyses performed before and after microsurgical repair of varicocele. All analyzes were carried out in the same public andrology laboratory in Brazil from 2016 to 2019. The three most critical seminal parameters (concentration, motility and morphology) were compared between preoperative and postoperative. The parameters established by the WHO (world health organization) for the categories were considered: Oligospermia (sperm count ≤15 million/ml); Severe Oligospermia (sperm count ≤5 million/ml); Atenozoospermia (total motile sperm < 40%); and Teratozoospermia (normal forms <4%, according to the Kruger criterion). To realize the statistic analise, the Shapiro-Wilk normality test was applied to verify the adherence of continuous variables to the normal distribution. The descriptive analysis of the variables that obtained adherence to the normal distribution was performed by means and standard deviations (Mean ± SD). For variables that did not have a normal distribution, the median, the 25 and 75 percentiles were used. The analysis of categorical variables was performed using absolute and relative frequencies. The Student's t test for paired samples was applied to continuous variables that showed normality and the Wilcoxon test was applied to those that did not show normality. Fisher's exact test was used to analyze the association between categorical variables. The significance level of 5% was adopted for all analyzes.
**Results**: The separate analysis of each seminal parameter showed that the number of patients (four) who after surgery were upgraded from the category Oligospermia Severa to Oligospermia or from Oligospermia to Normal Concentration Standard, was the same number (four patients )who were downgraded from the category Oligospermia to Oligospermia Severa or Standard Normal to Oligospermia. The rest (12 patients) remained in the same category. The same situation happened in the assessment of motility parameter, the number of patients (two) who were upgraded from the category of Astenozoospermia to Normal Motility standard was the same (2 patients) who dropped from the category Normal Pattern to Astenozoospermia in the postoperative. Sixteen patients had no change in the category. About Morphology parameter, there was a small post-surgical advantage, six patients were upgarded from the Teratozoospermia category to Normal Morphology Standard, while four patients dropped from the Normal Morfology Standard to Teratozoospermia category after varicocelectomy. Ten patients remained in the same category.
**Conclusion**: In view of the results, we found that varicocelectomy did not have a clinically positive impact for any of the three seminal parameters analyzed. These findings reinforce that such procedure does not significantly contribute to improving male fertility.

## P-21. Blastocysts euploidy predictor: Standard morphology better than the speed of the embryo development

**A.L.O. Barbeitas[1], A.C.A. Mancebo[1], R.A. Antunes[1], M.M. Souza[1], P.C.F. Areas[1], M.C.B. Souza[1]**

**Objective**: This is a study to evaluate if standard blastocyst morphology and speed of development correlates with euploidy rates assessed by preimplantation genetic testing for aneuploidies (PGT-A) of trophectoderm biopsies after intracytoplasmic sperm infection (ICSI).
**Methods**: 428 embryos from 206 patients were genetically analyzed previously by array CGH (embryos biopsied in 2015) and afterwards by NextGen sequencing (embryos biopsied in 2016-2017). All genetics analysis were performed in the same laboratory. All ICSI/PGT-A cycles with conclusive results were included in this study performed in a single private center between December 2015 and December 2017. Embryos from infertile patients with PGT-A indication (more than two failed IVF cycles/ previous spontaneous abortion/ male factor) were biopsied on day 5 or 6 according to degree of expansion and quality of trophectoderm and inner mass cell (ICM). They were categorized in three groups: Group 1 (134 embryos): Not expanded but good blastocyst (2AA, 2AB, 2BA, 2BB); Group 2 (63 embryos): Expanded/ Not expanded blastocyst with one poor classification (2/3 AC, CA, BC, CB); Group 3 (231 embryos): Fully expanded blastocyst with good morphology (3AA, 3AB, 3BA, 3BB). Fisher exact test and ANOVA was used for statistical analyses. $p$ value <0.05 were considered statistically significant.
**Results**: There were no differences in general demographic characteristics and laboratory data of the patients studied (Table 1). Women's age varied from 27 to 48 years. Most embryos were biopsied on day 5 (61%) while on day 6 (39%). The overall euploid rates were 42.5%, 36.5% and 62.3% in groups 1, 2 and 3 respectively. Group 3 presented higher euploidy rates comparing with groups 1 ($p$<0.0003) and 2 ($p$<0.0003). In a comparative analysis on day 5 blastocysts versus day 6 blastocysts, no significant difference in euploidy rates were found in each morphological group (Table 2).

**Table 1.** Comparison of demographic characteristics and laboratory data

| | Group 1 | Group 2 | Group 3 | *p* Value |
|---|---|---|---|---|
| AGE (y) | 37.52±3.83 | 37.89±3.74 | 36.93±3.67 | 0.27 |
| BMI (Kg/m²) | 24.13±4.27 | 24.53±4.41 | 24.27±4.55 | 0.93 |
| AMH | 2.22±1.99 | 1.92±1.42 | 2.42±1.54 | 0.07 |
| DAYS OF OVARIAN STIMULATION | 9.45±1.60 | 9.98±1.17 | 9.80±1.77 | 0.29 |
| OOCYTES RETRIEVED | 11.63±6.30 | 12.00±6.69 | 11.93±5.91 | 0.92 |
| MATURE OO-CYTES | 8.64±4.42 | 9.00±4.99 | 8.94±4.42 | 0.91 |
| NUMBER OF EMBRYOS | 7.32±3.92 | 7.88±4.33 | 7.55±3.96 | 0.83 |

Data are presented as Mean ± SD

| **Table 2.** Euploidy rates comparing D5 vs D6 | | | |
|---|---|---|---|
| | **D5** | **D6** | ***p* Value** |
| Group 1 (n=134) | 42.3 | 43.4 | 1.000 |
| Group 2 (n=63) | 45.8 | 30.7 | 0.285 |
| Group 3 (n=231) | 64.5 | 59.6 | 0.495 |

Data are presented as number (%)

**Conclusion**: According to our findings blastocyst euploidy rates seem to be related to embryo morphology more than to the speed of development. Our study is limited by its retrospective nature. A higher sample size or a prospective randomized design could be used in future studies to corroborate the current findings.

## P-22. Sperm DNA Fragmentation and semen parameters do not predict embryogenic outcomes following Intracytoplasmic Sperm Injection (ICSI)

**E.P. Miranda[1,2], M. Lourenço[1], F. Tenório[3,4], A.M. Lima[1], E.G. Sá[1], G.S. dos Santos[1], J. de Bessa Jr.[5], E. Torquato[1]**

**Objective:** The goal of this study was to evaluate the impact of semen parameters and sperm DNA fragmentation (SDF) index on the outcomes of ICSI. It was also our aim to search for clinical and laboratorial predictors of favorable endpoints.
**Methods:** We performed retrospective review of ICSI cases in a single center from January 2019 through June 2020. All couples whose male partner had performed a semen analysis with SDF testing in our center were included. Sociodemographic and clinical parameters were obtained from medical records. Laboratorial analyses closest to the day of ICSI were considered for the purpose of this analysis. The following traditional seminal parameters were included in our statistical model: total sperm count, total motility, Kruger strict morphology, teratozoospermia index, sperm deformity index, and index of acrossomic normality. For the evaluation of chromatin integrity sperm chromatin dispersion assays (SCD - Halosperm) were utilized, and both SDF and degradation index (DI) were evaluated. Pre-implantation embryogenic parameters were observed and the main outcome measure was rate of blastocyst formation (RBF) among mature oocytes that were injected with sperm.
**Results:** There were 55 consecutive cycles from 37 couples that were eligible for review. The mean male age was 41.2± .5 while the mean female age was 36.7±4.0. Among men, 2.7% were smokers, 47.9% were sedentary, 70.2% referred alcohol consumption, 2.7% had obesity (IMC > 30), and 40.5% had varicocele. The following pre-implantation embryogenic parameters were found: fertilization rate of 0.93±0.35, cleavage rate of 0.76±0.31 and RBF 0.45±0.36. There was no significant correlation between any of the analyzed semen parameters and RBF, including SDF (Pearson r = 0.01, 95%CI -0.31 to 0.33, *p*= 0.95). A subgroup analysis considering men with low SDF (<20%) demonstrated a higher blastulation (56% vs. 46%), which did not reach statistical significance (*p* = 0.4).
**Conclusion**: Seminal parameters and SDF were not significant predictors of better embryogenic outcomes during ICISI in our study population. Of note, clinical and laboratorial parameters were also not statistically associated with more favorable results.

## P-23. Intravenous Lipid Emulsion use in Assisted Reproduction Techniques: Is it related to higher pregnancy and live birth rates?

**V.C. Dornelles[1], M.R. Hentschke[1], L.M. Burmann[1], R.G. Petracco[1], V.D. Trindade[1], A.T.F. Kira[1], Talita Colombo[1], Alvaro Petracco[1], Mariangela Badalotti[1]**

**Objective**: To evaluate pregnancy rate after intravenous lipid emulsion (ILE) use in assisted reproduction techniques (ART) in patients with implantation failure history.
**Methods**: Retrospective cohort study performed at an assisted reproduction clinic in Brazil, including 359 patients, with at least two failure embryos transfer previously. The data refers to a period from April/2017 to February/2019 and were collected from electronic records. Samples were divided into two groups: Group 1, who underwent ILE infusion (n=64) and Group 2, who did not receive ILE (n=295). Group 1 received 2 ml of 20% ILE infusion, and after biochemical pregnancy confirmation, some patients received a second and/or third dose of the medication, according to medical indication. Data were presented as mean±standard deviation or frequency and percentage. For statistical analysis, Student's test or Fisher's exact test, and Pearson correlation were used, considering *p*<0.05.
**Results**: Comparing ILE group vs. Control group, the following results were found: maternal age (36.9±4 vs. 36.6±3.7, *p*=0.711), paternal age (40.3±5.3 vs. 39.9±6.3, *p*=0.474), biochemical pregnancy (50% vs. 41.5%, *p*=0.141), clinical pregnancy (45.3% vs. 37.6%, *p*=0.158). Regarding clinical outcomes, the rates of abortion/ectopic pregnancy was 9.3% vs. 2.0%, *p*=0.010 and birth rate was 35.9% vs. 35.6%, *p*=0.53. When gestational age, Apgar index, birth weight and length were analysed, no statistical differences were found. From the 45.3% clinical pregnancies in ILE group, 69% received 2 or more doses of the medication, leading to a positive correlation between ILE doses and clinical pregnancies variables (r=0.669, *p*<0.001). When comparing those who received one ILE dose with who received two or more doses, a higher birth rate, biochemical and clinical pregnancy rate were observed when more than one dose was used (*p*<0.001).
**Conclusion**: In the present study, ILE was not related to higher pregnancy and live birth rates; however, its use could be an option for couples undergoing ART with more than two implantation failures. Moreover, more than one dose of ILE should be encouraged when positive biochemical pregnancy is presented, in order to improve chances of clinical pregnancy and birth rate.

## P-24. The increase in Body Mass Index negatively affects sperm motility

**I.B. Teloken[1,2], C. Teloken[1,3], A. Arent[1], A. Petracco[1], M. Badalotti[1,2]**

**Objective:** Evaluate the impact of body mass index (BMI) on semen analysis results in patients seen at an infertility practice.
**Methods**: Observational, cross-sectional, retrospective study using data from patients seen at a private infertility clinic between 2010 and 2020. All the sperm analyses were performed in the same lab, according to the 2010's parameters of the World Health Organization. The first semen analysis from each patient was selected. Patients with a history of cancer treatment, cryptorchidism, viral orchitis, altered karyotype, microdeletions, vasectomy reversion, and testosterone use were excluded. Possible interfering factors were analyzed – age, smoking, use of alcohol and drugs, use of medication, physical activity, systemic diseases, and scrotum heat factors (saunas and immersion baths) – and adjustments were made for the ones that had p<0,10 in bivariate analysis. Student t-tests was used for means comparations and linear multiple regression analysis was used to identify associations.
**Results: F**rom a total of 1384 patients, 219 were excluded, and data from 1147 individuals were analyzed. The BMI varied between 18.9 and 50.8 kg/m². From the 1147 patients, 297 had a healthy weight (BMI between 18.5 and 24.9 kg/m²), 611 were overweight (BMI between 25 and 29.9 kg/m² ), 179 had obesity class 1 (BMI between 30 and 34.9 kg/m² ), 42 had obesity class 2 (BMI between 35 and 39.9 kg/m² ) and 18 had obesity class 3 (BMI greater or equal to 40 kg/m²). Due to the small number of patients, we put groups 4 and 5 together. The comparison of the groups seminal parameters shows a significant decrease in progressive and total motility in patients with BMI ≥35 ($p$=0.007 and $p$=0.012, respectively) (Table 1).
The complementary analysis determined BMI 29 kg/m² as the cutoff for impact on progressive motility ($p$=0.044) and 31 kg/m² on total motility ($p$=0.036). The results were still significant after age, use of cannabis and hypertension adjustments – the other possible interfering factors were not significant.

**Table 1.** Sperm parameters according to BMI

| | Healthy weight | Overweight | Obesity class I | Obesity classes 2 and 3 |
|---|---|---|---|---|
| N | 297 | 611 | 179 | 60 |
| Volume (ml) | 3.2 | 3 | 2.9 | 2.9 |
| Concentration (num/ml) | 45.6 | 45.8 | 45.9 | 42.2 |
| Total number | 138.9 | 124.1 | 121.8 | 99.9 |
| Vitality (%) | 80.2 | 80.3 | 81 | 80.3 |
| Progressive Motility (%) | 43.9 | 44.1 | 42.4 | 35.3* |
| Total Motility (%) | 54.5 | 54.1 | 53.4 | 46.1** |
| Normal forms (%) | 4.6 | 4.5 | 5.2 | 5.1 |

*$p$=0.007
**$p$=0.012

**Conclusion:** Our data show a reduction of sperm motility with increasing BMI, with impairment starting on 29 kg/m² for progressive motility and 31 kg/m² for total motility. In this study, all other semen parameters (volume, sperm concentration, vitality and morphology) were not impacted by obesity. Our data support the potential negative role of increasing BMI on male fertility. Since obesity is a major health problem of increasing prevalence worldwide, we remind the importance of weight control in infertility prevention.

## P-25. The euploidy rate in embryos from a single public center

**C.A.Z. Nissel[1], V. Heinrich-Oliveira[1,2], P.A.A Monteleone[1,3], E.C. Baracat[4]**

**Objective:** This study aims to demonstrate euploidy and aneuploidy rates among embryos from patients undergoing assisted reproductive treatment.
**Methods**: In the present study we analyzed prospectively the data of every patient which were randomized in groups for Preimplantation Genetic Testing for aneuploidy (PGT-A) in a major prospective randomized trial which compares the successful rate of a single elective transfer associated with PGT-A. All patients were submitted to the same controlled ovarian stimulation, with a short protocol using recombinant Follicle-stimulating Hormone (FSH) at a dosage of 225 IU in the first 5 days of the stimulus followed by at least another 4 days at a dosage of 150 IU. The blockage of luteinizing hormone (LH) surge was realized with Gonadotropin-Releasing Hormone (GnRH) antagonist and, the trigger was with a GnRH agonist. After the retrieval, every oocyte was submitted for intracytoplasmic sperm injection (ICSI). Patients with at least two good quality blastocysts, according to the Gardner scale, were randomized into two groups. A trophectoderm biopsy was performed in every embryo from the PGT-A group and the sample was analyzed by next generation sequencing. All embryos were submitted to the freeze-all strategy.
**Results:** Seven couples were included in this analyze, with an average female age of 31,28 years. The other characteristics of the studied population are shown in the table below (table 1).
From the 148 retrieved oocytes, 126 were mature (M II), which resulted in forty-six biopsied and analyzed good quality embryos, from which twenty-four were D5 blastocyst and twenty-two D6 blastocyst. Sixty-five percent of the analyzed embryos was euploid and 20,93 percent was aneuploid. Thirteen percent of those blastocysts had insufficient DNA sample or an inconclusive analyze and requires a new biopsy. From the 30 euploid embryos, 20 reached the blastocyst stage and were biopsied at day five (D5) and other 10 did it at day six (D6), there was significative statistical difference in the euploidy rate between D5 and D6 embryos ($p$=0.0124). All couples obtained at least one euploid embryo for transfer.

**Table 1.** Characteristics of the studied population

| CHARACTERISTICS OF PATIENTS | | 95% IC |
|---|---|---|
| Age (female partner) (years) | 31.28 | 28.8–33.74 |
| Age (male partner) (years) | 35.28 | 31.35–39.22 |
| Time of Infertility (months) | 42.42 | 16.9-67.9 |
| BMI (kg/m$^2$) | 22.47 | 20.65-24.29 |
| Average Number of Oocytes Retrieved (n) | 22.14 | 10.67-33.60 |
| Infertility Diagnosis | | |
| Tubal factor (%) | 57.14% | 4/7 |
| Uterine factor (%) | 14.29% | 1/7 |
| Endometriosis (%) | 14.29% | 1/7 |
| Genetic factor (%) | 14.29% | 1/7 |
| Male factor (%) | 57.14% | 4/7 |

**Conclusion:** Even good quality embryos from young patients, when submitted to genetic analysis, have an aneuploidy rate that cannot be ignored. In addition, karyotype defects become much more frequent in embryos that reach the blastocyst stage only on the sixth day of development. Unfortunately, due to the sample size, it was not possible to observe whether there are other factors correlated with the rates of aneuploidy in the studied population. Finally, we know that genetic analysis is an useful device aiming to reduce the time to pregnancy, however in some cases there is no clear indication for increasing the cost of treatment with the use of this technology or it isn't available. In these cases, knowing the behavior of embryos in the studied population is of great importance. The results of this paper can be a great weapon in the choice of embryos to be transferred according to their characteristics, showing which group has a higher prevalence of euploid embryos in this population. Future analyzes with a larger number of patients and embryos may reinforce the results obtained and bring new information to assist clinical practice.

## P-26. Dichorionic Diamniotic twins after a single blastocyst transfer in a frozen/thaw cycle following PGT-A

**R.A.Antunes[1,2], M.C.B. de Souza1, M.M. de Souza[1], A.C.A. Mancebo[1], V.A. Raupp1, B.M.L de Melo[2]**

**Introduction:** Multiple pregnancies are a reality in assisted reproductive cycles, being almost 20 times more common compared to the naturally conceived. However, there was a reduction of the twins' birth rate after elective single embryo transfer (eSET). Accordingly to embryology, all twins derived from an eSET should be monochorionic, either diamniotic, when the division occurs between the 4th and 8th day, or monoamniotic after the 8th day. Dichorionic diamniotic twins should be the result of an embryo splitting within 3 days.

**Objective:** Report a case of dichorionic, diamniotic twin pregnancy after an eSET.

**Case Report**: Case description:35 years old woman submitted to a bilateral salpingectomy, arrived to perform an IVF. Husband had a normal semen analysis. There was no history of multiple pregnancy in either families. She had 1 IVF cycle in another clinic and 4 embryo transfers (ET) between 2012-2017, without success. In 2017, the couple decided to go through another IVF cycle and preimplantation genetic test for aneuploidy screening (PGT-A). It was administered an antagonist protocol (Cetrotide®) with 2100UI of recombinant FSH + 1050UI of recombinant LH (Pergoveris®). Oocyte maturation trigger was a GnRH agonist (gonapeptyl daily®). The protocol resulted in 10 oocytes, which 8 were in metaphase 2. Intracitoplasmatic spermatozoid injection was the fertilization technique used. 7 oocytes had fertilized into 2 embryos. They were cultured individually in 20 µl droplets of CSCM-C under a layer of light mineral oil (Irvine Scientific®) in a Benchtop triple gas incubator (COOK®) until day 5. The blastocysts were frozen using vitrification kits (Ingamed®). PGT-A of the 6 blastocysts was performed by next generation sequencing technique (Igenomix®), and 3 male embryos were euploid. Both 1st and 2nd ET of 1 blastocyst resulted in negative pregnancy test. An endometrial receptivity test (ERA test®) was performed, presenting a normal result. The last ET was in 2018. On the day of the transfer of the last ET an image of him totally hatched and almost completely divided was registered. After 12 days she presented a BHCG of 2573mUI/mL. 2 weeks later, 2 distinct gestational sacs, with visible embryos and heart activity were detected. The patient underwent a cesarean section, giving birth of 2 male newborns, weighing 3025 and 2560 grams.

**Comments**: Discussion :This case serves as proof that the mechanism of monozygotic twinning is flawed. It is possible to have dichorionic, diamniotic twins from a single 5th day blastocyst. In our case, a natural pregnancy is not possible since the patient is salpingectomized. Monozygotic twins are rare after SET regardless the stage of the transferred embryo. Kyono *et al*. (2011) and Knopman *et al*. (2014) suggested extended culture as an explanation of monozygotic twinning. They implicated that embryos from oocytes younger than 35 years and assisted hatching also increased it. Van Langedonckt *et al*. (2000) reported a half blastocyst herniated through the zona pellucida that after transfer developed into 2 gestational sacs with fetuses, but no heart activity. Behr & Milki (2003) reported a full blastocyst splitting in day 6, therefore it was not preserved, nor transferred. Shibuya & Kyono (2012) reported the birth of monozygotic, dichorionic, diamniotic twins after the transfer of a single, previously frozen blastocyst. In none of those previous embryo trophectoderm biopsy was performed. The report of dizygotic, dichorionic twins as the result of a SET in a salpingectomized patient proves that twinning process is not fully understood.

## P-27. Enzymatic digestion improves testicular sperm retrieval

**A.M. Lima[1], G.S. Santos[1], SE Torquato Filho[1], E.P. Miranda[1].**

*[1]BIOS – Centro de Medicina Reprodutiva do Ceará*

**Objective:** To increase the sperm recovery success of micro dissection testicular sperm extraction (MICROTESE), with enzymatic digestion if no sperm is obtained from testis tissue by mechanical method.
**Methods:** Tissue samples were collected from 21 men who presented with clinical and laboratory data indicating NOA by MICROTESE. Initially, mature spermatozoa were examined in laboratory for by mechanical extraction technique. In cases whom no spermatozoa was observed after maximum 50 minutes of initial searching under the inverted microscope, the procedure was followed by enzymatic digestion using DNase I and collagenase type IV (DN 25- 10mg, Sigma and C5138- 25 mg, Sigma) diluted in modified HTF (IRVINE) supplemented with 10% of albumin (IRVINE). After incubation at 37$^{o}$C for 1 hour, the resultant solution was centrifuged for 5 minutes at 50g to remove remaining tissue residues. Diluting the supernatant with fresh medium, two more washing steps were conducted sequentially. The final pellet was re-suspended in the medium and the presence of free spermatozoa was checked under the inverted microscope.
**Results:** Of 21 cases with NOA, conventional mincing method extended with enzymatic treatment yielded successful sperm recovery in 06 (about 28.5%) patients.
**Conclusion:** The combination of MICROTESE and enzymatic digestion is an effective method to recover spermatozoa. The benefit of the mincing combined with enzyme to sperm retrieval for NOA firstly shorten the mechanical searching time, leading to minimizing further cellular damage as well as exposure to external conditions, and secondly reduce the number of cases with sperm recovery failures.

## P-28. The effect of polycystic ovary syndrome on oocyte and embryo quality in women submitted to in vitro fertilization

**G.S. Santos[1], A.M. Lima[1], S.E. Torquato Filho[1], E.G. Sá[1], S.C.S. Santiago[2].**

**Objective:** To investigate, through literature review, whether the metabolic and reproductive changes present in polycystic ovary syndrome (PCOS) can affect the development and quality of oocytes and embryos.
**Methods:** Integrative literature review, based on the analysis and comparison of data from specific studies, such as: rates of maturation, fertilization, implantation, and clinical pregnancy, which showed changes present in PCOS.
**Results:** Six studies were selected for a more careful analysis, in which it was found that the presence of obesity concomitant with PCOS can affect the development of oocytes and embryos and reduce rates of maturation and fertilization. In addition, hyperinsulinemia and hyperandrogenism can affect the quality of oocytes and embryos and promote a reduction in implantation rates and clinical pregnancy.
**Conclusion**: Through the analysis and comparison of data from the selected studies, it was possible to find evidence that some disorders intrinsic to PCOS can affect oocyte quality and embryonic development, hindering the success of the IVF procedure. There is a need for too many studies to show this approach.

## P-29. Thyroid autoantibodies in ovarian follicular fluid: is there an impact in assisted reproductive technology?

**D.C.S. Bastos[1], M.I. Chiamolera[2], R.E.C Silva[2], M.C.B. Souza[3], R.A. Antunes[3], M.M. Souza[3], A. C. A. Mancebo[3], P.C.F. Arêas[3], F.M. Reis[4], F. F. Bloise[1], T. M. Ortiga-Carvalho[1]**

**Objective**: The thyroid autoimmunity (TAI) is the most prevalent autoimmune disease in reproductive age women and is related to infertility and recurrent miscarriage. The presence of thyroid peroxidase autoantibodies (TPO-Abs) and thyroglobulin antibodies (Tg-Abs) in the serum characterizes TAI. Several studies have shown an association of TAI and infertility, even when TAI patients present normal thyroid hormone levels. Therefore, suggesting that the possible cause of fertility reduction in women with TAI is not only related to the hormonal imbalance. Recently, thyroid autoantibodies (TAA) were found in the follicular fluid (FF) of women with TAI, suggesting a direct role of TAA in female infertility. Herein, we analyzed if there is an association between TAA levels in FF and serum. Also, we investigated whether TAI impact assisted reproductive technology (ART) parameters.
**Methods**: This prospective study enrolled 61 women, 36.0±0.4 years, range 25-43, undergoing ART injection in two different reproductive centers, one with 39 and the other 22 patients, for three years. Patients were classified into two groups: those TAI positive (n=38, 35.5±0.6 years) and TAI negative without the disease (n=23, 36.7±0.6 years). Blood samples were drawn before the ovarian stimulation for TAA levels measurement. FF was obtained on the oocyte pick up day, the fluid was frozen and kept until the moment they were sent to be analyzed for TPO-Abs and Tg-Abs levels at the same laboratory, under the Elecsys (ROCHE) and homemade kit, respectively. We analyzed the general characteristics of patients undergoing ART and related information. The number of oocytes retrieved, number of oocytes MII per patient, fertilization rate, number of embryos of good quality obtained was compared between the two groups. The good quality embryo was considered the grade A embryo with 6 to 8 cells and <10% fragmentation on day 3. From the total patients in the study (61), 8 underwent egg freezing. Thus, the fertilization rate was analyzed only in 53 women,being 35 TAI positive and 18 TAI negative. Grade of embryos rate were obtained only in one reproductive center with 39 women, excluded who did egg freezing (TAI positive=13, TAI negative=17).
**Results**: The TAI positive group showed higher levels of TPO-Abs in serum (64.1±15.4 vs. 10.6±1.4IU/mL, $p$=0.15) compared to TAI negative group. We observed an increase in FF of Tg-Abs (3815±601 vs 148±22IU/mL, $p$<0.0001) and TPO-Abs levels (93.7±11.0 vs. 9.2± 1.0IU/mL, $p$=0.01) TAI positive compared to TAI negative group. Besides that, the two groups had no significant differences in serum TSH ($p$=0.89) and free T4 ($p$=0.53) levels. Although slightly higher in TAI positive group, we did not observe a significant difference in the rate of previous miscarriages (26.3% vs. 17.3%, $p$=0.53). Number of oocytes retrieved (9.2 vs. 8.6, $p$=0.93) was similar between the two groups. In TAI negative the mature oocyte (76.5% vs. 81.8%, $p$=0.47) and fertilization rate (75.0% vs. 87.0%, $p$=0.08) were slightly higher but also not different. Also, the grade A embryos rate (57.0% vs. 65.3%, $p$=0.53) was little higher in the TAI positive group.
**Conclusion**: We demonstrated the presence of high levels of TAA in the FF of women with TAI. We did not see any differences in the analyzed parameters. However, we cannot ignore the fact that number of analyzed patients is still low. Therefore, our result highlights a possible role of TAA in ART parameters. Further evidence is required to clarify whether the cross-reaction of these antibodies with ovarian targets contributes to the poor outcome of the ART in these patients.

## P-30. The impact of advancing male age on the Assisted Reproduction procedures: laboratorial and gestational outcomes

**B. Cunegatto[1], M. Badalotti[1], M.R. Hentschke[1], A. Petracco[1], C.F. Ferreira[2], R.M. Azambuja[1], C. Telöken[1], M.T.V. Sanseverino[1], M.F. Jimenez[2]**

**Objective:** Male reproductive aging may affect several determinants in assisted reproduction techniques outcomes. The literature usually considers the age of 40 as a limit. After this period seems to have changes in qualitative factors that may decrease fertility and increase adverse events in the future generation. This study aim is to evaluate if advanced male age is associated with unfavorable outcomes on assisted reproduction techniques.
**Methods:** A retrospective cross-sectional study was conducted using a database from an assisted reproduction clinic in southern Brazil, with anonymized information, between January 2015 and December 2018. A total of 2125 patients met the eligible criteria. The sample was divided into two groups according to male age: group 1 (n=1429), ≤ 40 years and group 2 (n=696), ≥ 41 years. This study included couples who sought care for infertility treatments. The Intracytoplasmic sperm injection technique was performed in all cases. Mann-Whitney test, Student's t-test and Chi-square test were applied for statistical analysis. Additionally, statistical adjustments for some variables of interest were made by "female age" and "vasectomy", using Logistic Regression or Covariance Analyzes (ANCOVA), when applicable. The null hypothesis was rejected when $p$<0.05.
**Results:** A decrease in seminal volume and sperm motility was observed as the male age increased ($p$=0.001 for both parameters), with no changes in seminal concentration ($p$>0.05). In addition, a higher number of embryos were transferred on the third day of development in group 2, while a higher number of embryos was transferred on the fifth day, in group 1 ($p$≤0.001). Apgar index in the first and fifth minutes were also lower in group 2 for single and twin pregnancies, even after adjusting by female age ($p$<0.05). The number of indications for assisted reproduction techniques due to oligospermia was higher in Group 1, while the indication due to vasectomy occurred more in Group 2. For the variables: rate of fertilized oocytes, embryonic blastulation and euploidy, pregnancy rates, live births rate and other neonatal outcomes, no differences were found between groups, after statistical adjustment by female age.
**Conclusion:** Advanced male age appears to be a possible influential factor on seminal characteristics, specially sperm volume and motility. Hence, even though previous literature points out female age as the main IVF outcome influencer, male age should not be left aside.

## P-31. Introduction of Time-Lapse incubator in the laboratory: A strategy to validate for clinical use

**R. Azambuja[1], B. Cunegatto[1], M.R. Hentschke[1], A. Ribeiro[1], F.M. Wingert[1], V.C.[1]; Trindade, V.D. Dornelles[1], A. Petracco[1], M. Badalotti[1]**

**Objective**: To present a strategy to validate the introduction of time-lapse technology in the laboratory for clinical use.
**Methods**: Retrospective case-control study performed at an assisted reproduction clinic in southern Brazil. The data refers to a period from January to February 2020 and was collected from electronic records. Inclusion criteria: patients undergoing *in vitro f*ertilization (IVF), with at least two fertilized embryos. A total of 39 patients were included in the analysis. Embryos from the same patient were then divided into two groups: Group 1 (G1), embryos cultured in the Time-lapse technology and Group 2 (G2): embryos cultured in a conventional incubator. A total of 417 inseminated embryos (G1=210; G2=207) and 188 blastocysts (G1=111; G2=77) were included. Fertilization rate, embryo development and blastocyst rate were analyzed between groups. Variables were expressed in median [25th-75th]. Mann–Whitney U-test test was applied, and statistical significance was defined as $p$<0.05.

**Results**: The mean maternal age was 34.6±4.4. When compared G1 with G2 the following results were observed: inseminated embryos (5 [4-6] *vs.* 5 [4-7], *p*=0.800); fertilized embryos (5 [3-6] *vs.* 4 [3-5], *p*=0.334); fertilized embryos rate (fertilized/inseminated) (83.3% [71.4-100.0] *vs* 83.3 % [66.7-100], *p*=0.657); embryos on day 3 with more than 6 cells (4 [2-5] *vs.* 2 [1-5] (*p*=0.028)); number of blastocysts (embryos on day 5 plus day 6) (3 [1-4] *vs.* 1.5 [1-3], *p*=0.039) and blastocysts rate (60.0% [33.3-75.0] *vs* 45% [18.6-61.7], *p*=0.038).
**Conclusion**: In this study, data from 39 patients and 188 blastocysts was sufficient for demonstrating statistical difference regarding to embryo development in different incubators. Hence, the applied methodology in this study may be a strategy to validate the introduction of time-lapse technology in the laboratory for clinical use.

## P-32. PCOS: Myo-inositol combined to aromatases inhibitors and ovulation induction: a better choice?

**V.A. Raupp[1], L.B.A. Silva[1], J. Reis[1], A.C.A. Mancebo[1], M.M. Souza[1], R.A. Antunes[1], M.C.B.Souza[1]**

**Objective:** Polycystic ovary syndrome (PCOS) is the most common cause of female infertility due to reproductive and metabolic implications. Usually there results insulin resistance and compensatory hyperinsulinemia with an increased ovarian androgen production along with the reduction of hepatic sex hormone binding globulin(SHBG) production. Myo-inositol (MYO), one of the nine stereoisomers of Inositol, has been reported to significantly decrease hyperandrogenism and insulin resistance in women with PCOS, including restoring spontaneous ovarian activity and thus fertility in these patients. So, the objective of this study was to compare a combination of letrozole and myo-inositol or letrozole in ovulation induction of infertile women with PCOS.
**Methods**: Prospective longitudinal randomized single center study. From Nov 18 to March 2020, women ≤39years of age with infertility and PCOS as defined by the Rotterdam criteria. Participants were selected in a 1:1 ratio according to acceptance, to either 5,0 mg letrozole (Letrozol®, EurofarmaSP) on cycle days 2-6 up to 3 treatment cycles, or combined to Myo-inositol 2g + folic acid 200mcg (Fertisop®, Stragen Pharma, SP), 4g sachets, used in the dose of one sachet every 12 hours, minumum period of 4 weeks previously to LTZ. Exclusion criteria: tubal obstruction, uncorrected thyroid disease or hyperprolactinemia, sperm count<15 million/mL and motility A+B≤32%. Baseline data included weight, height, BMI, blood pressure, characterization of menstrual cycles (R- regular I-irregular, A- secondary amenorrhea). Ovulation was monitored with transvaginal ultrasound from D10 of the cycle, at most with follow-up till D20, looking for the presence of 1 to 3 follicles ≥16mm, with endometrial thickening ≥7mm, characterizing the pre-ovulatory response. Responses ≥ 4 follicles of this order would result in guidance for abstaining from intercourse due to the risk of multigemelarity. The decision to inject hCG to trigger ovulation was made by the attending physician. Couples were advised to have at least one intercourse from the pre-ovulatory/HCG day up to 3 days. In 4 days, confirmed the presence of corpus luteum, micronized progesterone or dihydrogesterone was started for 10 days. Statistics: Chi-square test was used at a two- sided significant level of 0.05 for differences between the two groups.
**Results:** We intended 31 patients in each arm to obtain a 80% statistical power to demonstrate a clinical 33% absolute difference between the groups based on the PPCOS II trial.There resulted 20 patients in the Group LTZ-Myo and 19 patients in the LTZ group. And, 6 of these last 19 patients in fact added Metformin- MTF (500-1500mg/day) to their protocols, after their endocrinologists or OBGyn, before starting LTZ (Table 1). Among the patients with MYO + LTZ, 6 had regular cycles, 10 irregular and 4 were amenorrheic. Among those with LTZ, 12 had regular cycles, 6 irregular and one was amenorrheic. The combination LTZ-Myo was not different from LTZ alone (or associated to MTF) during the three ovulation induction cycles (Table 2). We had a high ovulation rate in both arms (94% x 94%), mostly mono pre-ovulatory follicles. There resulted one pregnancy with LTZ+Myo (first cycle, ongoing) x two pregnancies with LTZ alone, one ongoing and the other 1st trimester miscarriage. Three other patients were pregnant from 3 to 8 months of only Myo (ongoing) while one other got pregnant without LTZ or MTF, ongoing.

**Table 1.** Patients Characteristics

| | Patients Characteristics | | |
|---|---|---|---|
| | MYO + LETROZOL (N =20 ) | LETROZOL (N=19 ) | *p* VALUE |
| AGE | 33.90 ± 2.24 | 33.78 ± 2.46 | 0.88 NS |
| WEIGHT | 67.30 ± 14.54 | 75.37 ± 10.90 | 0.11 NS |
| HEIGHT | 165.4 ± 5.22 | 167.15 ± 6.30 | 0.41 NS |
| BMI | 24.90 ± 5.58 | 27.04 ± 4.39 | 0.27 NS |
| AMH | 5.74 ± 4.86 | 5.63 ± 7.64 | 0.95 NS |
| HB GLICADA | 5.09 ±0.32 | 5.30 ± 0.39 | 0.16 NS |
| HOMA - IR | 2.21±1.28 | 1.78 ± 0.78 | 0.34 NS |

Values Means ± SD

**Table 2.** Number of cycles

**Table 2.** Number of cycles

| | Number of cycles (N patients) | | | |
|---|---|---|---|---|
| Groups | 1 Cycle | 2 Cycles | 3 Cycles | Total |
| Myo-Inositol + LTZ (N =16 patients) | 5 (5) | 8 (4) | 21 (7) | 34 (16) |
| Letrozol (N=16 patients) | 3 (3) | 16 (3) | 15 (5) | 34 (16) |
| | | | | 68 (32) |

**Conclusion:** Although this study did not fulfill the initial proposed numbers to compare Myo-inositol as an adjunct to LTZ in ovulation induction and LTZ alone (or associated to metformin), there was no difference in the ovulation results. It still remains opened myo-inositol qualities, but 4 amenorrhoeic patients recovered ovulation during the induced cycles with Myo-LTZ, while other three spontaneous pregnancies occurred with just myo-inositol, ongoing.

## P-33. Clinical and lab evaluations in "Dual Stim" protocol for poor responders

**E. Araújo Filho[1], L.P. Araújo[1], M.E. Pavan[1], C.L. Fácio[1], L.A. Machado-Paula[1], L.F.P Araújo[1]**

**Objective:** To compare the two phases of the ovarian stimulation in the dual stim protocol in poor responders in terms of number of follicles, number of international units (IU) of gonadotrophin, number of oocytes, mature oocytes, fertilization, cleavage and blastocyst rate.
**Methods:** Prospective observational study performed from January/2019 to February/2020 in a cohort of poor responders (Poseidon 3 and 4), that underwent IVF with the dual ovarian stimulation protocol for recruting and increased number of oocytes in the same cycle. A total of 25 patients were included in the study. The protocol of stimulation was the same described Ubaldi *et al*. (2016).
**Results:** Patients age ranged from 34-42 years (average 39 years). The Anti-Müllerian hormone (AMH) ranged from 0.1 to 0.8 (average 0.33). The average IU of gonadotrophin in the first follicular stimulation was 2050 and in the second follicular stimulation 1600IU ($p$=0.4). The average number of follicles in the first stimulation was 4.2 and in the second stimulation 5.5 ($p$=0.2). The average of mature oocytes recovered in the first stimulation was 2.0 and in the second stimulation was 2.6 ($p$=0.09). The normal average fertilization rate in the first stimulation was 1.4 and in the second 2.2 ($p$=0.09). The average cleavage stage day 3 embryos in the first stimulation was 1.0 and in the second stimulation 1.6 ($p$=0.13) and the average number of blastocyst grade A and B frozen in the first phase of the stimulation was 1.0. In the second stimulation the number of blastocysts frozen was 1.3 ($p$=0.25).
**Conclusion:** The double ovarian stimulation showed a second ovarian stimulation with oocytes, with the same fertilization rate, cleavage, and the blastocyst rate, tending to be higher than the first stimulation in poor responders. Our study showed a similar response in the first and second stimulation of the dual stim protocol, with a tendency to have more mature and fertilized oocytes in the second stimulation. The dual stimulation may increase the number of oocytes and embryos in poor responders (Poseidon 3 and 4).

## P-34. Association between MTHFR mutation and antiphospholipid antibodies with poor ovarian reserve

**L.P. Araújo[1], M.E. Pavan[1], L.F.P. Araújo[1], C.L. Fácio[1], L.A. Machado-Paula[1], E. Araújo Filho[1]**

**Objective:** Methylenetetrahydrofolate reductase (MTHFR) gene variants are associated with lower ovarian reserve, diminished response to follicular stimulation and a reduced chance of live birth rate after IVF. Our study evaluates the incidence of MTHFR mutations and anti-phosphatidylserine (aPS) and anti-phosphatidylethanolamine (aPE) of infertile patients with an AMH less than 0.8 ng/ml.
**Methods:** This is a prospective observational cohort study of infertile patients undergoing infertility investigation in the period of January/2019 to March/2020. A total of 98 patients with an AMH level under 0.8 ng/ml were included in the study, with less than 38 years of age. However, 50 patients all the work out exams to be included in the study.
**Results:** In the study group (AMH less than 0.8), 37 patients out of 50 had a level of anti-phosphatidylserine greater than 20.0 (IgG, M, A), which gives an incidence of 75% of aPS/aPE in this group of patients. Fourty six out of the 50 patients included in the study tested for mutations in the MTHFR and 27 had it positive, which gives an incidence of 59%.
**Conclusion:** We observed a high incidence of mutation in the MTHFR enzime in patients with very low AMH (less than 0.8 ng/ml) under 38 years of age. We also observed a high incidence of acquired thrombophilya (aPS/aPE) in this group of patients.

## P-35. Ovarian hyperstimulation syndrome after a frozen-thawed blastocyst transfer. A case report

**M.G. Cequinel[1], C.A. Cornel[1], T.C.S. Bonetti[2,3]; P.A.A. Monteleone[2,4]**

**Objective:** Ovarian hyperstimulation syndrome (OHSS) is an iatrogenic complication which occur in the luteal phase and/or early pregnancy after use of ovulation inducing agents. It is a life-threatening condition and cycle cancellation, ovulation triggering with GnRH agonist and freeze-all embryos must be considered as prevention when risk is observed. Although OHSS after spontaneous pregnancy is rare, it has been reported in women with hypothyroidism, polycystic ovarian syndrome (PCOS), molar pregnancy, pituitary adenomas and mutations in FSH receptors. This is a case report a severe OHSS after frozen- thawed embryos transfer (FET) in a woman with chronic anovulia.
**Case report:** A couple with primary infertility, 26 years-old woman, secondary amenorrhea, hormones and ultrasound (US) suggesting PCOS and 31 years-old man with normal semen analysis. Started ovarian stimulation with Letrozole 5mg/day for programmed intercourse cycle in September 2018. Cycle was cancelled due to follicles diameter were <10mm. A second cycle was started in October 2018 with Letrozole 5mg/day plus r-FSH 75IU and 10 follicles (17-20 mm) were observed in the 11º day. Trigger was performed by GnRH agonist and 24 oocytes were collected. A total of 23 MII oocytes were fertilized by ICSI with ejaculated sperm of partner, 21 oocytes had normal fertilization and 12 blastocysts developed. Six good quality blastocysts were biopsied for PGT-A and cryopreserved. Four embryos were euploid. A hormone replacement cycle (November 2018) was performed and one euploid blastocyst was transferred with a β-HCG negative outcome. A second FET was performed in a hormone replacement cycle (February 2019) and two euploid blastocysts were transferred. A positive β-HCG (395 IU/mL) was observed and clinical pregnancy with two gestational sacs was confirmed by US. At 9 weeks of pregnancy (March 22, 2019), US showed ongoing pregnancy of twins, increased ovaries (right ovary (RO): 223 $cm^3$, left ovary (LO): 143 mm3), bilaterally stimulated follicles (20mm) and β-HCG of 269000.00 IU/ml. Patient was oriented to maintain vaginal progesterone each 12hrs and rest. Patient got into the hospital on March 31, 2019 due to pain and ascites. A paracentesis was performed with improvement of clinical symptoms and discharge after 2 days. On April 03, US revealed ongoing pregnancy of 11 weeks, increased ovaries (RO 750cm3 and LO 603$cm^3$) and ascitis (600cm3). Two days after, patient got into intensive care unit (ICU) due to clinical worsening and three paracentesis were performed in the following days (4.5L, 4.0L and 3.0L). β-HCG was 235895.00IU/ml. Hospital discharge on April 18, 2019 when US showed twin ongoing pregnancy of 13 weeks, increased ovaries (OD 1207cm3 and LO 939cm3), ascitis (350$cm^3$) and β-HCG of 228226.00IU/ml. The clinical condition of patient was stable and was followed by symptomatology and US. A cesarean section was performed at 36 weeks of pregnancy and two female live babies born.

**Conclusion:** A severe OHSS occurred after FET and resulted in hospitalization for more than 3 months and premature birth. Ovarian augmentation continued until the end of the pregnancy. Of greater interest in the present case, although OHSS with spontaneous pregnancy is rare, twin pregnancies characterized by elevated β-hCG levels, enlarged ovaries, ascites and other physical findings suggestive of intravascular depletion may be the result of OHSS. Early diagnosis and supportive care are essential in the resolution of a potentially lethal syndrome. Finally, we suggest that single embryo transfer may decrease the likelihood of developing severe OHSS in cases where freezing all embryos is used to prevent OHSS.

## P-36. Mayer-Rokitansky-Kuster-Hauser Syndrome: report of 2 cases of patients with the syndrome who underwent assisted reproduction treatment

**C.A.G. Santos[1], J.P.B.M. França[1], A.P.S. Aguiar[1], B.A.B. Scheffer[1], R.F.C.B. Scheffer[1], J.A.B. Scheffer[1]**

**Introduction:** In this article, 2 cases of Mayer-Rokitansky-Küster-Hauser Syndrome (MRKH) patients who made assisted reproduction treatment at our service will be reported on. The first with embryo transfer to the gestational carrier and the second with embryos freezing.

**Case Report 1:**34-year-old patient, already diagnosed with the syndrome, sought the service in August, 2018 for treatment. Patient reported left oophorectomy, neovagina surgery and rudimentary uterus. She took her sister to be the gestational carrier. Examinations were requested from the couple and the uterus donor, where the patient was the value of 3.58 of the Antimuller Hormone (AMH). In June of 2019, the first ovarian stimulation was started with the use of recombinant FSH. In the transvaginal ovular collect were removed six eggs and all of them are mature (MII). ICSI (intracytoplasmic sperm injection) was performed with the partner's semen, 4 eggs were fertilized with good development and fresh transfer to the sister's uterus (after simultaneous endometrial preparation) of 2 blastocysts - both embryos with B classification. There were no embryos left to freeze. The beta-HCG test result was negative. The couple opted to try again and Performing DuoStim was proposed. Patient restarted in August of 2019 and ovarian stimulation with recombinant FSH and LH. 13 eggs were collected transvaginally and all were vitrified. The second stimulus was started 5 days after collection using the same medication. Five more eggs were collected and frozen. In December of 2019, after the endometrial preparation of the patient who gave up the uterus (her sister), the eggs were thawed and 17 survived. All were injected with the partner's semen and using the ICSI technique. 13 eggs were fertilized, and on the 5th day it was observed the presence of 1 class A embryo, 2 class B and 6 class C embryos - the others were blocked. Transfer of 2 blastocysts and freezing of 4 embryos were done. Beta-HCG test result was positive and the US demonstrated the presence of 1 gestational sac and an embryo with a heartbeat.

**Case Report 2:** 33-year-old patient, already diagnosed with MRKH's syndrome and sought the service in May of 2019 for treatment. The couple wanted to freeze embryos, since they had no one to give up a uterus for pregnancy at the time. Examinations were requested from the couple and her AntiMullerian Hormone (AMH) was value of 5.4.In June, 2019 ovarian stimulation was started with application of recombinant FSH and LH. In the transvaginal ovular collect of 27 eggs, 19 of them were mature. ICSI was used with partner's semen and fertilization of 11 out of the 19 eggs. On the third day, 8 embryos were frozen: 2 class A, 4 class B, and 2 class C embryos. The couple has frozen embryos and they haven't transferred yet.

**Comments:** In the cases performed at our service, it was observed that the patients were young, with good ovarian reserve. A satisfactory amount of eggs was captured, with fertilization within the expected rates. The development of the embryos also showed no abnormalities. In vitro fertilization with the use of a gestational carrier is currently the most viable and effective way of obtaining live births for patients with MRKH syndrome. More studies are still needed to assist in the best techniques and protocols, however. In the future, uterine transplantation may be the treatment of choice.

## P-37. Importance of morphological classification of morula in its different stages of compaction correlation with pregnancy rates, in fresh transfers in IVF/ICSI cycles

**J.P. Rocha[1], M.C.C.F. Finotti[1], G.C. Borges[1], R.S. Florêncio[1], M.N.C.R. Camarco[1], V.A. Oliveira[1]**

**Objective**: Since the beginning of the studies in Assisted Reproduction, the best embryo has always been sought. Embryo transfers during cleavage stage (day 2 or day 3) and blastocyst stages (day 5 or day 6) are common in current daily practice in fresh IVF/ET cycles. Data regarding transferring day 4 embryos, morula/compact stage, is still restricted and the grading system is also inconsistent, as between IVF clinics. This study provided the key importance of the classification system for morula/ compact stage embryos and compared with successes pregnancy rates and ongoing rates.
**Methods**: This was a retrospective study. A review of medical records from January 1st, 2015, to December 31st 2019, performed for all conventional insemination and ICSI cycles with a GnRH antagonist or GnRH agonist protocol. It was realized a revision on morula classification transferred in four types (M4: ≥80% of compactation; M3: 60-80% of compactation; M2: 30-50% of compactation; M1: expanded and/or 20% of compactation) in two groups of age (group 1: ≤35 years and group 2: 36-40 years old). For transfer, we utilized our proper classification of transfer (type I: at least one M4; Tipo II: mórulas M3 and/or at least one M4; Tipo III: mórulas M2 and/or at least one M3; Tipo IV: mórulas M1 and/or at least one M2) of Humana Medicina Reprodutiva, Goiânia,Go, Brazil.
**Results**: In this study there were 236 cycles with fresh transfer included in our study, with transfer of 505 morulas (day 4). We finding in the Two groups: Group 1 (≤35years old), the following results of pregnancy: chemical, clinical, ongoing in % (Type I : 54.7, 46.1, 27.2; Type II: 45.6, 36.9, 24.4; Type III: 6.2, 0.0, 0.0; Type IV: 13.6, 13.6, 13.6). There results in Group 2 (36-40 years old) are: ( Type I : 65.5, 55.1, 36.0; Type II: 40.7, 32.0, 20.0; Type III: 37.5, 25.0, 25.0 ; Type IV: 14.81, 14.8, 7.41). However, when we compared the Type IV group with Type I and II, with in group 1 , we found a statistical difference ($p$<0.05).
**Conclusion:** In conclusion, we stand out that this classification for morula stage transfer(D4) had a statistical difference between Type I versus Type IV and Type II versus Type IV in the chemical, clinical and ongoing pregnancy rates in two aged groups.

## P-38. Could the embryo biopsy be replaced by non-invasive assessment of the embryo?

**L.M.A. da Costa[1], I.P. Mamede[1], P.G.M. da Silva[2], R.V. Borba[2], S.A. de Oliveira[3]**

**Objective:** The evaluation of chromosomal status of embryos performed by the preimplantation genetic test through trophoectoderm biopsy (PGT-A) is currently the most reliable method of embryo selection. Nevertheless, trophoectoderm biopsy is considered an invasive assessment method, raising doubts about the risks for the embryo. Therefore, the genetic evaluation through nucleic acid from free cells in the blastocele fluid and the media that involves the blastocyst (cf-DNA), became an option of great clinical interest as it is non invasive, less challenging and more economical. The aim of the present study is to perform an updated review of the agreement rate between the noninvasive preimplantational study and the trophoectoderm embryo biopsy.
**Methods:**The present study is a review performed initially by active research on database of PubMed from 2014-2020. The following descriptors were selected: "cf-DNA", "PGT-A", "Trophoectoderm biopsy", "noninvasive", and their Portuguese translations. Two independent reviewers evaluated the available studies. The inclusion criteria were the description of the noninvasive method in comparison to the traditional PGT-A. The exclusion criteria were: studies that did not report about cf-DNA. At the pre-selection phase, 683 studies were found with the descriptors. After the selection, 8 articles met the elegibility criteria and were selected.
**Results**
The agreement rates ranged from 30.4% to 87.5% (Table 1).

**Table 1.** Selected studies and their concordance rates

| Article | Agreement rate of the methods | Notes |
|---|---|---|
| Vera-Rodriguez *et al*. Hum Reprod. 2018 | 30.4% | 113 embryos and 28 other control samples. Assessment performed on D5. Culture medium volume of 25 μL. |
| Capalbo *et al*. Fertil Steril. 2018 | 37.5% | 46 samples. Assessment performed on days D5, D6, and D7. Culture medium volume of 25 μL. |
| Yeung *et al*. J Assist Reprod Genet. 2019 | 62.1% | 168 samples. Assessment on days D5 and D6. Culture medium volume of 30 μL. |
| Ho *et al*. Fertil Steril. 2018 | 65.0% | 45 embryos, 20 of which became blastocysts. Assessments on D3 and D5. Culture medium volume of 25 μL. |
| Feichtinger *et al*., Reprod Biomed Online. 2017 | 72.2% | 22 blastocysts. Assessment performed on D5. Culture medium volume of 25 μL. |
| Rubio *et al*. Fertil Steril. 2019 | 78.7% | 108 blastocysts. Agreement rate went up to 84% when the noninvasive assessment were performed on D6 or D7. Culture medium volume of 10 μL. |
| Xu *et al*. Proc Natl Acad Sci U S A 2016 | 85.7% | 42 blastocysts. The embryos on D3 were frozen under vitrification and warmed up and cultured at 37°C for 2 days. Culture medium volume of 30μL. |
| Kuznyetsov *et al*. PLoS One. 2018 | 87.5% | 28 blastocysts. Assessment performed on D5 and D6. Culture medium volume of 25μL. |

**Conclusion:** The agreement rate from the noninvasive method and the PGT-A were considered satisfactory in the majority of the studies, supporting the choice of cf-DNA. However, a variation between the rates was considerable requiring more research for a better assessment and conclusion about this subject.

## P-39. Is the use of pentoxifylline in the selection of epididymal or testicular spermatozoa still worth it?

**C.P. Pompeu[1], T.V. Feitosa[1], L. Vellez[1], I.H. Yoshida[1], M.M. Gava[1], E.B. Cordts[1], CP Barbosa[1]**

**Objective:** To evaluate the effectiveness of the use of pentoxifylline (PTX) in stimulating immotile epididymal or testicular spermatozoa, previously to the intracytoplasmic sperm injection (ICSI), and to compare the rate of fertilization, cleavage, blastocyst formation and pregnancy rates with the control group.
**Methods:** The retrospective study included patients who had sperm motility induced with pentoxifylline after percutaneous aspiration of the epididymis (PESA), percutaneous aspiration of the testis (TESA) or testicular biopsy (TESE) and later ICSI (n = 22); and patients who underwent the same surgeries and subsequent ICSI, but without the use of pentoxifylline (n = 22), from January 2019 to March 2020. Trained embryologists performed the evaluation of fertilization and embryo development. Embryo transfer (ET) was performed on the third day of development (D3) or in the blastocyst stage. Some embryos were cryopreserved for later TE. After TE, the concentration of β-hCG in the blood was measured. Statistical analysis was performed using the chi-square test, considering a *p* value less than 0.05 statistically significant.
**Results:** Patients undergoing PESA, TESA or TESE and later ICSI were divided into two groups, using PTX (n=22) and control (n=22), and then had their clinical results compared. The ages of women (34.2±5.17, with PTX *vs*. 34.1±5.83, control) and men (40.5±6.25 *vs*. 43.7±10.0), showed no statistically significant difference between both groups, as well as the blastulation rate (33.8% *vs*. 34.0%) and the pregnancy rate (58.3% *vs*. 31.3%), respectively. The fertilization rate (89.5% *vs*. 71.5%) was significantly higher in the group using PTX when compared to the control group, but the cleavage rate (70.8% *vs*. 98.1%) was significantly lower in the group using PTX than in the control group.
**Conclusion:** The effectiveness of the motility enhancement by pentoxifylline has beneficial effects and as long as used correctly, there are no negative implications for its applicability.

## P-40. Dichorionic and diamniotic pregnancy after intracytoplasmic sperm injection associated with viable fetus and fetus with chromosomal alteration compatible with Patau syndrome at 31 weeks: case report

**M.N.C Rocha[1], F.C. Santos[1], R.S.S.F. Ghiggi[1], A.A. Araujo[1], M.P. Batista[1], M.R. Borges[1]**

**Introduction:** Patau syndrome is caused by a chromosomal abnormality, known as trisomy of 13.Its cause is the non-disjunction of chromosomes during anaphase 1 of mitosis, generating gametes with 24 chromatids. Studies on the relationship between intracytoplasmic sperm injection (ICSI) and the incidence of trisomy 13 are scarce in the literature. Advanced paternal age may be related to low sperm quality, but little is known about its effect on rates of aneuploid embryos and chromosomal abnormalities such as trisomy 13.
**Case report:** O.B. F., third pregancy, 40 years old, with Marfan Syndrome, with a previous abortion and two twin pregnancies after treatment by reproductive medicine. Two in vitro fertilizations were performed by male factor (oligospermia and varicocele). In the third pregnancy, dichorionic and diamniotic gestation, ultrasound changes were observed in fetus 1: holoprosencephaly alobar, proboscis and interventricular communication. Diagnostic confirmation of chromosome 13 trisomy, compatible with Patau syndrome, was obtained after amniocentesis. The ultrasound follow-up detected, at 31 weeks of gestation, intrauterine growth restriction of the malformed fetus. At 31 weeks and 05 days, the patient had gestational hypertension, being hospitalized and diagnosed with early pre-eclampsia. During hospitalization, the fetus 1's fetal beats were not founded and one day after that his amniotic membranes ruptured. The second fetus continued with good fetal vitality. She was submitted, at gestational age of 31 weeks and 05 days, to a cesarean section due to maternal indication, due to the clinical worsening of the preeclampsia. During the surgical procedure, hemoamnium and placental detachment of approximately 40% were observed. There were no complications in the puerperal period and the healthy baby evolved well after intensive neonatal treatment.
**Comments:** Currently, using assisted reproduction technology, to select the best embryos morphologically, it is possible to accomplish preimplantation genetic testing invasive and non-invasive. The main indications are still found among women with advanced age, recurrent abortions, implantation failure and history of personal and / or family genetic diseases. However, it is not yet unanimous among the most Brazilian centers to biopsy all cases of infertile patients. Morphological analysis remains a good methodology in the choice of embryos eligible for embryos transfer in most cases. This case stands out for presenting after two embryos transfer resulting in a dichorionic and diamniotic twin pregnancy with a viable fetus and at home after 31 weeks of gestation and another with chromosome 13 trisomy with phenotypic characteristics of Patau syndrome. There are few reports in the literature of a pregnancy with this trisomy evolving in a successful gestational age for the second fetus.

## P-41. Assessment of infertility factors in patients seen at a Reproduction outpatient clinic at Hospital Regional Leste, in the Federal District, from March 2016 to March 2018

**F.B. Uchôa[1], L.S. Côrtes[2], V.L. Lima[2], MWM Dias[2]**

**Objective:** The main objective is to assess the factors of infertility prevalent in patients seen at a Reproduction outpatient clinic of the Hospital Regional Leste of the Federal District, from March 2016 to March 2018. The specifics, on the other hand, refer to the assessment of the incidence of female and male infertility factors taking into account the parameters of age, type and time of infertility of the patients; referral rates for assisted reproduction services, after the couple's infertility diagnosis and comparison of the rates of these infertility factors obtained with data of scientific evidence.

**Methods**: This article was part of the work to conclude the Medical Residency in Obstetrical Gynecology by one of the authors. It was submitted and approved by the Research Ethics Committee of FEPECS - SES-DF (Foundation for Teaching and Research in Health Sciences of the State Health Secretary of the Federal District), whose CAAE number is 97778918.0.0000.5553. This is a quantitative, descriptive, retrospective and observational study. The data were obtained from the analysis of electronic medical records, using the anamnesis of the first consultation to collect information regarding age, time of union with the partner and infertility; menstrual, obstetric and sexual history (including sexual dysfunction and frequency of intercourse; surgical and gynecological history; use of medications; family history, including family members with infertility; investigation of smoking and alcohol consumption, as well as questioning the age of the partner , history of toxic habits (alcoholism and smoking), type of job occupation and primary or secondary infertility.

**Results:** 246 medical records of patients seen at the Human Reproduction Clinic of the HRL were analyzed. However, only 52.03% of medical records had conclusive information regarding the investigation of infertility factors. Thus, the sample consisted of 128 patients, seen between March 2016 and March 2018. The mean age of the patients was 34.12, with a minimum age of 19 years and a maximum of 47 years, with standard deviation ± 6.70. Therefore, 55.46% of the patients belonged to the age group of 31 to 40 years old and 17.96% were older than 40 years. The study showed 58.6% of primary infertility and 41.4% of infertility secondary and when considering the couples' sterility time, it is noticed that the great most were between two and five years, reaching a percentage of 65.5%. The study showed 58.6% of primary infertility and 41.4% of infertility. In the series of the present study, 58.59% of women had regular cycles, 39.84% had irregular cycles and 1.56% were amenorrhea. The absence of menstruation in women of childbearing age can be justify by numerous factors, and for this small sample of the study (1.56%) the cause established was hyperprolactinemia, which needs to be investigated and its cause defined, to later consider the possibility of a pregnancy. Talk about pain during intercourse (dyspareunia) and previous history of illness pelvic inflammatory disease (DIPA), the importance of a thorough clinical history is perceived. The data showed that 62% of the patients did not report discomfort or pain during sexual intercourse and 38% of them had dyspareunia, regardless of their position assumed in the sexual act. When considering that the main cause of infertility due to tuboperitoneal factor is the most occurrence of DIPA, it is clear that the incidence of such pathology in the sample was 18.75. In this study, 51.56% of couples have frequency sexual intercourse three times a week, which theoretically would be a necessary condition for conceiving a pregnancy in couples that do not have any impediment to such. To a lesser extent, 19.53% and 15.62% of couples have sexual frequency of four and twice a week, respectively. The analysis of the age group showed that 5.4% of the patients' partners less than 25 years old. Among these, none showed any change in the spermogram or had to undergo an additional diagnostic test for completion of the couple's infertility factor. In the same percentage, 5.4% had older than 46 years, and among these 71.42% had changes in the spermogram, they are then responsible for the couple's infertility factor. The majority of men, 49.21%, were between 36 and 45 years old and the second largest portion, 38.28%, were between 26 and 35 years old, with the average age between they are 36 years old.

## P-42. How old it's too cold ? Live birth with sperm cryopreserved for 17 years.

**A. Arent[1,2], C. Teloken[1], I. Teloken[1], D. Farinatti[1], T. Colombo[1], R. Azambuja[1], A. Petracco[1], M. Badalotti[1,2]**

**Objective**: to describe a successful pregnancy and live birth after in vitro fertilization (IVF) cycle using a semen sample cryopreserved for 17 years.

**Case report:** A 16-year-old male teenager, in 2001, was diagnosed with a testicular embryonal carcinoma. He was referred to our fertility Center and 3 seminal samples were colected in 3 consecutive days, prior to cancer treatment (right orchiectomy and chemotherapy), resulting in 12 ampoules of cryopreserved sperm. In 2013 he was diagnosed with a testicular seminoma and a left orchiectomy was performed, with no more possibility to conceive naturally.

In 2018 he and his 33-year-old female partner looked for infertility treatment. An IVF cycle was run using the cryopreserved semen. The frozen/thawed semen quality was similar to the fresh basal sample (9 million/ml with 33% of motility). A total of 10 mature oocytes were inseminated by intracytoplamatic sperm injection (ICSI), and 8 were fertilized, resulting in 4 blastocysts embryos. One blastocyst was transferred to the uterus and 3 were vitrified. The pregnancy was confirmed and she delivered a healthy boy, with 39 weeks of gestational age, weighing 3600 g.

**Comments**: There are very little reports in the medical literature on successful pregnancies using human semen stored for a long time. The first report being from the UK after 21 years of storage. However, many regulatory authorities set a time limit on sperm (and egg) storage of ten years. This case report provides evidence that long-term freezing can successfully preserve sperm quality and fertility.

## P-43. Does embryo morphology in different maternal age groups interfere with the clinical pregnancy rates in frozen euploid blastocysts transfers?

**I.H. Yoshida[1], E. Souto[1], C.H. Suganuma[1], M.S. Tanada[1], C.Z. Berton[1], E.B. Cordts[1,2], C.P. Barbosa[1,2]**

**Objective:** Verify the pregnancy rate of euploid blastocysts by associating their morphological quality to different maternal ages in frozen transfer cycles.
**Methods:** Cross-sectional study was performed in the period from 2016 to 2019 where which information on frozen transfer cycles with PGT-a was collected. 1230 embryos were biopsed and genetic analysed. Clinical pregnancy rates were evaluated according to their morphology and to the maternal age. The transferred euploid blastocysts were split into two groups: Top Quality group (TQ), with ratings: AA, AB, BA; and Not Top Quality group (NTQ) with ratings: BB, BC, CB and CC. Maternal age was assessed and segregated into 3 groups: patients up to 35 years old; between 36 and 39 and over 40. The data were evaluated and statistical analysis was performed using the Chi-square test.
**Results:** Of the 1230 embryos analyzed, 850 showed aneuploidies (69.1%) and 380 were euploid (30.9%). The TQ blastocysts formation showed no statistical difference between maternal age groups, no matter what the genetic status of the embryos were. The pregnancy rate in TQ euploid blastoscysts group was higher when compared to the NTQ group ($p$ = 0.001); but showed no statistical significance among different maternal age groups. When euploid transfers were stratified in different age groups, it was observed that the morphological quality had no influence on the patients over 40 years group ($p$ = 0.278).
**Conclusion:** The combination of both parameters, embryo morphology and genetic chromosomal analysis, showed to be more effective to access embryo quality, resulting in higher pregnancy rates, no mattering patient age. It can be an important tool to achieve a single, healthy pregnancy in the shortest possible time.

## P-44. Metformin: anovulation treatment and improvement in the reproductive profile of patients with polycystic ovarian syndrome

**S.N.B. Baptista[1], G.M. Teixeira[1], N.G. Araujo[1], M.G. Pinheiro[1].**

[1]*Universidade Santo Amaro*

**Objective:** To associate the effects of Metformin therapy with the treatment of anovulation and improvement of the reproductive profile in women with Polycystic Ovarian Syndrome (PCOS).
**Methods:** Students from a private medical school in São Paulo conducted a bibliographic review of articles published from 2010 to 2020 related to databases such as Pubmed, Scielo and Cochrane. The research prioritized clinical trials, randomized or not, and systematic reviews, besides being limited to Portuguese, English and Spanish languages. The search in the databases was performed using the following descriptors: Metformin; polycystic ovarian syndrome; anovulation; female infertility and reproduction, registered in the Health Sciences Descriptors (DeCS).
**Results:** After analysing the characteristics of each of the 15 articles studied on the subject of interest, it is remarkable the improvement with metformin-based therapy in anovulation and reproductive profile of women with Polycystic Ovarian Syndrome (PCOS). This occurs because MTF has as direct effect the inhibition of steroidogenesis of the theca cells, and indirect in the contribution of the improvement of insulin resistance, reducing the androgenic production of the thecal cells. Thus, its administration contributes to the regulation of hyperinsulinemia, with a reduction in the circulating level of androgens associated with increased production of sex steroid-bearing globulin (SHBG) and control of the menstrual cycle of women with PCOS. Therefore, MTF improves the chance of reestablishing ovulation, reflecting in higher pregnancy rates and live births. This fact is evidenced in the studies analysed, for example, in 2016, when a randomized clinical trial (RCT) was carried out, selecting 100 patients with PCOS, non-obese and with normal sensitivity to insulin. These patients were monitored for 6 months, resulting in an ovulation rate of 100% for those who used metformin, compared to 37% for those who were monitored with placebo. Regarding the progression of pregnancy rate and live births, was observed in another prospective randomized, double-blind study, a 12-week treatment before and during in vitro or ICSI fertilization in non-obese women with PCOS, demonstrating that the use of metformin was effective in relation to pregnancy and live birth rate when compared to placebo administration, achieving a 16.7% difference in improvement.
**Conclusion:** MTF therapy establishes an ovulatory pattern in patients with PCOS. Acting both in improving insulin resistance and in reducing the androgenic production of thecal cells, the use of MTF provides an increase in the rate of pregnancy and live births in patients, thus ensuring a better reproductive profile for women with PCOS.

## P-45. Progestin-primed ovarian stimulation is an effective protocol for oocyte or embryo cryopreservation cycles: a cohort analysis and review of literature

**M.S. Siqueira[1], A.S. Amaral[1], M.E. Amaral[1], A.C.P. Barbosa[1], N.P. Barbosa[1], J.R. Iglesias[1], H.M. Nakagawa[1], M.W.P. Barbosa[1]**

**Objective**: To determine the efficacy of progestin-primed ovarian stimulation protocols.
**Methods:** We performed a retrospective cohort analysis regarding patients that underwent controlled ovarian stimulation in a private assisted reproduction clinic, between January 2019 and December 2019. Inclusion criteria was use of progestin-primed ovarian stimulation (PPOS) protocols, in women submitted to oocyte cryopreservation or in vitro fertilization (IVF). The primary outcome was number of metaphase II (MII) oocytes retrieved. Secondary outcomes were number of oocytes retrieved and duration of stimulation. We also performed a systematic review to identify relevant randomized clinical trials (RCT) that evaluated the efficacy of PPOS protocols.
**Results:** A total of 24 women were included: 15 were submitted to oocyte cryopreservation and 9 to IVF. Average age was 35,5 and AFC was 13,3; number of oocytes and MII oocytes retrieved were 10,0 and 7,5, respectively; duration of stimulation was 10,5 days. There were 3 cases of premature luteinizing hormone (LH) surge, with ultrasonographic evidence of corpus luteum during the oocyte pick-up. Regarding the systematic review, the last electronic search performed on June 10, 2020 retrieved 12 records. Eight studies were considered eligible and included in the narrative review.

**Conclusion:** Despite the few number of cases analysed in our cohort, there is evidence from RCTs suggesting that PPOS protocols provide similar reproductive outcomes compared to conventional protocols. Besides the efficacy, other aspects such as costs and ease of use should be evaluated when choosing a controlled ovarian stimulation protocol. In this way, progestins could be considered a reasonable option for preventing the LH surge.

## P-46. Profile of couples attended in a public-private reproduction center

**C.P. Chiariello[1,2], T.A. Viana[1,2], R.L.B. Souza[1,2], J.C. Jayme[1,2], L.M. Labadessa[1,2], A.K. Bartmannn[1,2]**

**Objective:** There are assisted reproduction centers in Brazil that can be public, generally linked to university, private, and public-private, in which part of the treatment is paid by the couple themselves. The Ana Bartmann Human Reproduction Center in partnership with the University of Ribeirão Preto (UNAERP) offers free consultations, but with treatments paid by the couples. The service offered - with the presence of students in a university hospital that conducts research and with the ability to deal with complex cases - makes not all couples choose it. The profile of the public seeking this type of center is very specific. Therefore, in this study, we evaluated the profile of patients who seek and who, mainly, perform paid treatments in a university setting.

**Methodos:** Clinic's database analysis of the last 400 patients seen at the center until January 2020.

**Results:** Of the 400 selected patients, only 135 (33%) underwent the proposed treatment. The average age of women who underwent consultation was 35.3 years, and who underwent treatment 38.2 years. One hundred and fifty-one (37.75%) lived in the city of Ribeirão Preto, 90 (22.5%) in cities less than 100 km from the reproduction center and 159 (39.75%) more than 100 km. The main cause of infertility was low ovarian reserve. A small percentage of patients were oocyte donors (5.5%) and similarly, a small percentage of patients were oocyte recipients (7%). A considerable portion of the patients did not even receive a budget for the cost of treatment (60%), having attended only the initial consultations.

**Conclusion:** The study concluded that the main obstacle to treatment is its cost. However, the presence of students and residents and the lack of a logistical structure that facilitates the arrival, as well as the coming and going of patients can be factors of withdrawal. The fear of exposure to a group of students, of the eventual impossibility of bearing the costs of treatment are psychological factors that can and should be addressed by the reproductive center psychologist in a navigation scheme, as already existing in oncology centers. Older patients or those with more complex diagnoses generally accept a paid university service.


Carneiro M.M. 1 2
Koga C.N. 1
Mussi M.C.L. 1
Fradico P.F. 1
Ferreira M.C.F. 1 2
1 Departamento de Ginecologia e Obstetrícia, Faculdade de Medicina da UFMG
2 Centro de Reprodução Humana Mater Dei, Belo Horizonte, MG

P-04. Successful lyophilization of human spermatozoa confirmed by flow cytometry analysis of membrane integrity

Geber S. 1 2
Giraldo C.Q. 1
Bossi R.l. 2
Carvalho A.T. 3
Fernandes S.O.A. 4
Sampaio M. 2
1 Department of Obstetrics and Gynaecology of the Medical School, Universidade Federal de Minas Gerais, Brazil
2 ORIGEN, Center for Reproductive Medicine, Brazil
3 Integrated Research Group in Biomarkers, Rene Rachou Institute, Oswaldo Cruz Foundation - Brazil;
4 Department of Clinical Analysis, Faculty of Pharmacy, Universidade Federal de Minas Gerais, Brazil

P-05. The place reserved for man in reproduction treatments (in) fertility and (in) male visibility

Giacon F.A.S. 1
1 Psicóloga - especialização em Psicologia da Reprodução Humana, Instituto Suassuna de Goiânia - GO

P-06. The importance of validating new available technologies in laboratory: a study on the performance of a commercial software (Embryoscope/KIDScore™) to predict blastocyst implantation through scores subgroups evaluation.

Erberelli R.F. 1
Nicolielo M. 1
Jacobs C. 1
Mendez F. 1
Belo A. 1
Cremonesi L. 1
Paranhos I. 1
Motta E.L.A. 2
Lorenzon A.R. 3
1 Huntington Medicina Reprodutiva, Embryology, Sao Paulo, Brazil.
2 Huntington Medicina Reprodutiva/ Federal University of São Paulo, Clinical Director/Associate Professor, São Paulo, Brazil.
3 Huntington Medicina Reprodutiva, Research & Development, São Paulo, Brazil.

P-07. Analysis of morphokinetic parameters from blastocysts obtained from Progestin primed ovarian stimulation (PPOS) versus Antagonist protocol to inhibit LH surge. An experience of the time-lapse system in fresh egg donation cycles.

Aquino A.P. 1
Barros B.C. de 2
Calazans L. 3
Campos A.L.M. 4
Lorenzon A.R. 5
Motta E.L.A. da 6
Cury T.S.D. 7
1 Clinical Department - Vila Mariana Unit - Huntington Medicina Reprodutiva, São Paulo, SP, Brazil.
2 Embryology Laboratory - Vila Mariana Unit - Huntington Medicina Reprodutiva, São Paulo, SP, Brazil
3 Clinical Department - Pró-Criar Medicina Reprodutiva, Belo Horizonte, MG, Brazil.
4 Embryology Laboratory - Pró-Criar Medicina Reprodutica, Belo Horizonte, MG, Brazil.
5 Scientific Coordinator - Huntington Medicina Reprodutiva, São Paulo, SP, Brazil
6 Clinical Director - Huntington Medicina Reprodutiva, São Paulo, SP, Brazil
7 Egg donation Program Coordinator - Huntington Medicina Reprodutiva, São Paulo, SP, Brazil

P-08. Is there any pregnancy predict factors in patients who underwent Intrauterine Insemination? 13 years of experience in a public health University Hospital

Hentschke M.R. 1 2
Dornelles V.C. 1 2
Kobe L.M. 1
Cunegatto B. 2
Siqueira D.C. 1
Trindade V.D. 1 2
Furlanetti T.M. 1
Petracco A. 2
Badalotti M. 1 2
1 Pontifícia Universidade Católica do Rio Grande do Sul
2 Fertilitat – Centro de Medicina Reprodutiva

P-09. Morphokinetic Analysis of Blastocysts from frozen and fresh oocytes in Progestin Primed Ovarian Stimulation (PPOS) Protocol

Calazans L.C. 1
Campos A.L.M. 2
Moraes C.C. 2
Barros B.C. 3
Aquino A.P. 4
Lorenzon A.R. 5
Domingues T.S. 6
Caetano J.P.J. 7
1 Clinical Department - Pró-Criar Medicina Reprodutiva, Belo Horizonte, MG, Brazil.
2 Embryology Laboratory - Pró-Criar Medicina Reprodutica, Belo Horizonte, MG, Brazil.
3 Embryology Laboratory - Vila Mariana Unit - Huntington Medicina Reprodutiva, São Paulo, SP, Brazil
4 Clinical Department - Vila Mariana Unit - Huntington Medicina Reprodutiva, São Paulo, SP, Brazil.
5 Scientific Coordinator - Huntington Medicina Reprodutiva, São Paulo, SP, Brazil
6 Egg donation Program Coordinator - Huntington Medicina Reprodutiva, São Paulo, SP, Brazil
7 Clinical Director - Pró-Criar Medicina Reprodutiva, São Paulo, SP, Brazil

P-10. Dydrogesterone as an alternative to suppress LH surge in ART cycles

Souza M.C.B. 1
Mancebo A.C.A. 1
Souza M.M. 1
Antunes R.A. 1
Silva L.B.A. 1
Barbeitas A.L.O. 1
Raupp V.A. 1
1 Fertipraxis Centro de Reprodução Humana, Rio de Janeiro, RJ , Brazil

Table 1 Demographic and treatment characteristics.

 	CTA group (n=35)	DYG group (n=45)	p valor	
Age	36.40±4.00	38.26±2.68	0.01 SIGNIF	
Stimulation days	9.25±1.59	9.37±1.40	0.72  NS	
AMH	2.35±1.96	2.36±2.06	0.98  NS	
AFC	15.03±10.60	15.22±9.64	0.93  NS	
>16 < = 19	3.66±2.68	3.60±2.92	0.92  NS	
>= 20	2.26±1.67	3.02±2.04	0.07  NS	
BMI	25.36±4.41	23.48±4,23	0.07  NS	
LH at trigger	3.08±2.09	3.64±3.98	0.51  NS	
MII	6.71±5.73	7.55±5,31	0.49  NS	
MI	0.51±0.74	0.37±0.65	0.51  NS	
VG	1.80±2.24	1.64±2.98	0.51  NS	
Data expressed as averages ±SD

P-11. Clinical pregnancy in blastocyst transfer is correlated to blastulation morphokinetic parameter

Jacobs C. 1
Nicolielo M. 1
Mendez F. 1
Eberelli R. 1
Belo A. 1
Reis D. 1
Fauth C. 1
Motta E. L. A. 2
Lorenzon AR. 3
1 Huntington Medicina Reprodutiva, Embryology, Sao Paulo, Brazil.
2 Huntington Medicina Reprodutiva / Federal University of São Paulo, Clinical Director / Associate Professor, São Paulo, Brazil.
3 Huntington Medicina Reprodutiva, Research & Development, São Paulo, Brazil.

P-12. Graphic projection analysis as a psychological diagnosis tool in new family constitutions undergoing assisted reproduction treatment

Melamed R.M.M. 1
Braga D.P.A.F. 1 2
Borges Jr. Edson 1 2
1 Fertility Medical Group - São Paulo/SP
2 Associação Instituto Sapientiae - Centro de Estudos e Pesquisa em Reprodução Assistida - São Paulo/SP.

P-13. Morphokinetic Analysis of Blastocysts obtained after Progestin Primed Ovarian Stimulation (PPOS) and Double Stimulation (DUOSTIM)

Campos ALM 1
Calazans LC 1
Pereira LMR 1
Maia LMA 1
Amorim LVC 1
Guimarães BCL 1
Marinho RM 1
Caetano JPJ 1
1 Huntington/Pró-criar Medicina Reprodutiva

P-14. Stress and anxiety of women submitted to assisted human reproduction

Marchi B.R. De 1
Previato L.F. 1
Fácio C.L. 1
Machado-Paula L.A. 1
Araujo Filho E. 1
1 Centro de Reprodução Humana de São José do Rio Preto (CRH Rio Preto), São José do Rio Preto, SP, Brasil

P-15. Pregnancy rate in relation to embryo quality of fresh embryos in D2/D3 versus D5.

Branco A. Castelo 1
Fontes A.C.L. 1
Nachef S. 1
1 Art Fértil Clínica de Reprodução Humana, Recife, PE, Brasil.

P-16. Seminal fluid parameters and varicocele effects: the reality of a public service.

Silva U.B. 1
Lopes J.R.I. 1
Silva L.C.A. 1
Torres M.M.G. 1
Santanna A.L.B. 1
Martins M.Q.L.M. 1
Nóbrega E.J.P.B. 1
Barbosa K.R.N. 1
Aderaldo J.F. 1
1 Maternidade Escola Januário Cicco - UFRN - EBSERH- Natal/RN, Brazil.

P-17. Progestin Primed Ovarian Stimulation (PPOS) Protocol plus Double Stimulation (DUOSTIM) to inhibit LH peak: a plausible strategy for IVF cycles.

Maia L.M.A. 1
Caetano J.P.J. 1
Calazans L.C. 1
Pereira L.M.R. 1
Amorim L.V.C. 1
Campos A.L.M. 1
Moraes C.C. 1
Marinho R.M. 1
1 Huntington/Pró-criar Medicina Reprodutiva

P-18. Unveiling the surrogacy: the relationship between the baby´s biological parents and the “solidary belly” woman during and after pregnancy

Silva S.L.
Lopes R. C. S.
No institutional affiliation

P-19. Double Stimulation (DUOSTIM) for elective oocyte freezing: are follicular phase stimulation and luteal phase stimulation comparable?

Amorim L.V.C. 1 2
Pereira L.M.R. 1
Calazans L.C. 1
Maia LMA 1
Xavier E.B.S. 1
Campos A.L.M. 1
Olintho T.H.S. 1
Marinho R. 1
Caetano J.P.J. 1
1 Huntington/Pró-criar Medicina Reprodutiva

P-20. Effect of varicocelectomy on sperm quality in a public Andrology service in Brazil.

Lopes J.R.I. 1
Silva L.C.A. 1
Silva U.B. 1
Torres Torres M.M.G. 1
Santanna A.L.B. 1
Martins M.Q.L.M. 1
Nóbrega E.J.P.B. 1
Barbosa K.R.N. 1
Aderaldo J.F. 1
1 Maternidade Escola Januário Cicco -UFRN - EBSERH- Natal/RN, Brazil.

P-21. Blastocysts euploidy predictor: Standard morphology better than the speed of the embryo development

Barbeitas A.L.O. 1
Mancebo A.C.A. 1
Antunes R.A. 1
Souza M.M. 1
Areas P.C.F. 1
Souza M.C.B. 1
1 Fertipraxis - Centro de Reprodução Humana, Rio de Janeiro - Brasil.

P-22. Sperm DNA Fragmentation and semen parameters do not predict embryogenic outcomes following Intracytoplasmic Sperm Injection (ICSI)

Miranda E.P. 1 2
Lourenço M. 1
Tenório F. 3 4
Lima A.M. 1
Sá E.G. 1
Santos G.S. dos 1
Bessa Jr. J. de 5
Torquato E. 1
1 Centro de Reprodução Humana Evangelista Torquato
2 Universidade Federal do Ceará
3 Intituto de Medicina Integral Professor Fernando Figueira (IMIP)
4 Clínica Andros Recife
5 Universidade Estadual de Feira de Santana

P-23. Intravenous Lipid Emulsion use in Assisted Reproduction Techniques: Is it related to higher pregnancy and live birth rates?

Dornelles V.C. 1
Hentschke M.R. 1
Burmann L.M. 1
Petracco R.G. 1
Trindade V.D. 1
Kira A.T.F. 1
Colombo Talita 1
Petracco Alvaro 1
Badalotti Mariangela 1
1 Fertilitat - Center for Reproductive Medicine, Laboratory, Porto Alegre, Brazil

P-24. The increase in Body Mass Index negatively affects sperm motility

Teloken I.B. 1 2
Teloken C. 1 3
Arent A. 1
Petracco A. 1
Badalotti M. 1 2
1 Fertilitat - Centro de Medicina Reprodutiva, Porto Alegre.
2 PUCRS University.
3 UFCSPA University.

P-25. The euploidy rate in embryos from a single public center

Nissel C.A.Z. 1
Heinrich-Oliveira V. 1 2
Monteleone P.A.A 1 3
Baracat E.C. 4
1 Disciplina de Ginecologia - Departamento de Obstetrícia e Ginecologia. Hospital das Clínicas da Faculdade de Medicina da Universidade de São Paulo (FMUSP), São Paulo. Brasil
2 Divisão de Ginecologia Endócrina da Disciplina de Ginecologia - Departamento de Obstetrícia e Ginecologia. Hospital das Clínicas da Faculdade de Medicina da Universidade de São Paulo (FMUSP), São Paulo. Brasil
3 Centro de Reprodução Humana Monteleone. São Paulo. Brasil
4 Professor titular da Disciplina de Ginecologia - Departamento de Obstetrícia e Ginecologia. Faculdade de Medicina da Universidade de São Paulo (FMUSP), São Paulo. Brasil

P-26. Dichorionic Diamniotic twins after a single blastocyst transfer in a frozen/thaw cycle following PGT-A

Antunes R.A. 1 2
Souza M.C.B. de 1
Souza M.M. de 1
Mancebo A.C.A. 1
Raupp V.A. 1
Melo B.M.L de 2
1 Fertipraxis - Centro de Reprodução Humana
2 Universidade Federal do Rio de Janeiro (UFRJ)

P-27. Enzymatic digestion improves testicular sperm retrieval

Lima A.M. 1
Santos G.S. 1
Torquato Filho SE 1
Miranda E.P. 1
1 BIOS - Centro de Medicina Reprodutiva do Ceará

P-28. The effect of polycystic ovary syndrome on oocyte and embryo quality in women submitted to in vitro fertilization

Santos G.S. 1
Lima A.M. 1
Torquato Filho S.E. 1
Sá E.G. 1
Santiago S.C.S. 2
1 BIOS - Centro de Medicina Reprodutiva do Ceará
2 UNIFOR - Universidade de Fortaleza.

P-29. Thyroid autoantibodies in ovarian follicular fluid: is there an impact in assisted reproductive technology?

Bastos D.C.S. 1
Chiamolera M.I. 2
Silva R.E.C 2
Souza M.C.B. 3
Antunes R.A. 3
Souza M.M. 3
Mancebo A. C. A. 3
Arêas P.C.F. 3
Reis F.M. 4
Bloise F. F. 1
Ortiga-Carvalho T. M. 1
1 Institute of Biophysics Carlos Chagas Filho, Universidade Federal do Rio de Janeiro, Rio de Janeiro, RJ, Brazil.
2 Department of Medicine, Escola Paulista de Medicina, Universidade Federal de São Paulo, São Paulo, SP, Brazil.
3 Fertipraxis Centro de Reprodução Humana, Rio de Janeiro, RJ, Brazil.
4 Division of Human Reproduction, Department of Obstetrics and Gynecology, Universidade Federal de Minas Gerais, Belo Horizonte, MG, Brazil.

P-30. The impact of advancing male age on the Assisted Reproduction procedures: laboratorial and gestational outcomes

Cunegatto B. 1
Badalotti M. 1
Hentschke M.R. 1
Petracco A. 1
Ferreira C.F. 2
Azambuja R.M. 1
Telöken C. 1
Sanseverino M.T.V. 1
Jimenez M.F. 2
1 Fertilitat - Center for Reproductive Medicine, Laboratory, Porto Alegre, Brazil.
2 Federal University of Rio Grande do Sul (UFRGS), Department of Gynaecology and Obstetrics, Porto Alegre, Brazil.

P-31. Introduction of Time-Lapse incubator in the laboratory: A strategy to validate for clinical use

Azambuja R. 1
Cunegatto B. 1
Hentschke M.R. 1
Ribeiro A. 1
Wingert F.M. 1
Trindade V.C. 1
Dornelles V.D. 1
Petracco A. 1
Badalotti M. 1
1 Fertilitat - Center for Reproductive Medicine, Laboratory, Porto Alegre, Brazil.

P-32. PCOS: Myo-inositol combined to aromatases inhibitors and ovulation induction: a better choice?

Raupp V.A. 1
Silva L.B.A. 1
Reis J. 1
Mancebo A.C.A. 1
Souza M.M. 1
Antunes R.A. 1
Souza M.C.B. 1
1 Fertipraxis Centro de Reprodução Humana, Rio de Janeiro, RJ, Brazil.

P-33. Clinical and lab evaluations in “Dual Stim” protocol for poor responders

Araújo Filho E. 1
Araújo L.P. 1
Pavan M.E. 1
Fácio C.L. 1
Machado-Paula L.A. 1
Araújo L.F.P 1
1 Centro de Reprodução Humana de São José do Rio Preto (CRH Rio Preto), São José do Rio Preto, SP, Brasil

P-34. Association between MTHFR mutation and antiphospholipid antibodies with poor ovarian reserve

Araújo L.P. 1
Pavan M.E. 1
Araújo L.F.P. 1
Fácio C.L. 1
Machado-Paula L.A. 1
Araújo Filho E. 1
1 Centro de Reprodução Humana de São José do Rio Preto (CRH Rio Preto), São José do Rio Preto, SP, Brasil

P-35. Ovarian hyperstimulation syndrome after a frozen-thawed blastocyst transfer. A case report

Cequinel M.G. 1
Cornel C.A. 1
Bonetti T.C.S. 2 3
Monteleone P.A.A. 2 4
1 Embryo Centro de Reproduçao Humana, Curitiba. Brasil
2 Centro de Reprodução Humana Monteleone. São Paulo. Brazil
3 Departamento de Ginecologia. Escola Paulista de Medicina da Universidade Federal de São Paulo (EPM-UNIFESP), São Paulo. Brazil
4 Disciplina de Ginecologia - Departamento de Obstetrícia e Ginecologia. Faculdade de Medicina da Universidade de São Paulo (FMUSP), São Paulo. Brasil

P-36. Mayer-Rokitansky-Kuster-Hauser Syndrome: report of 2 cases of patients with the syndrome who underwent assisted reproduction treatment

Santos C.A.G. 1
França J.P.B.M. 1
Aguiar A.P.S. 1
Scheffer B.A.B. 1
Scheffer R.F.C.B. 1
Scheffer J.A.B. 1
1 Brazilian Institute of Assisted Reproduction (IBRRA), Belo Horizonte, MG, Brazil

P-37. Importance of morphological classification of morula in its different stages of compaction correlation with pregnancy rates, in fresh transfers in IVF/ICSI cycles

Rocha J.P. 1
Finotti M.C.C.F. 1
Borges G.C. 1
Florêncio R.S. 1
Camarco M.N.C.R. 1
Oliveira V.A. 1
1 Clínica Humana Medicina Reprodutiva, Goiânia, Go, Brasil.

P-38. Could the embryo biopsy be replaced by non-invasive assessment of the embryo?

Costa L.M.A. da 1
Mamede I.P. 1
Silva P.G.M. da 2
Borba R.V. 2
Oliveira S.A. de 3
1 Universidade de Rio Verde, Campus Aparecida de Goiânia, Aparecida de Goiânia, GO, Brazil
2 Faculdade de Medicina da Universidade Federal de Goiás, Goiânia, GO, Brazil
3 Departamento de Ciências da Vida da Universidade do Estado da Bahia, Salvador, BA, Brazil

P-39. Is the use of pentoxifylline in the selection of epididymal or testicular spermatozoa still worth it?

Pompeu C.P. 1
Feitosa T.V. 1
Vellez L. 1
Yoshida I.H. 1
Gava M.M. 1
Cordts E.B. 1
Barbosa CP 1
1 Instituto Ideia Fértil de Saúde Reprodutiva, Santo André/SP, Brasil.

P-40. Dichorionic and diamniotic pregnancy after intracytoplasmic sperm injection associated with viable fetus and fetus with chromosomal alteration compatible with Patau syndrome at 31 weeks: case report

Rocha M.N.C 1
Santos F.C. 1
Ghiggi R.S.S.F. 1
Araujo A.A. 1
Batista M.P. 1
Borges M.R. 1
1 Clínica Humana Medicina Reprodutiva, Goiânia, Go, Brasil.

P-41. Assessment of infertility factors in patients seen at a Reproduction outpatient clinic at Hospital Regional Leste, in the Federal District, from March 2016 to March 2018

Uchôa F.B. 1
Côrtes L.S. 2
Lima V.L. 2
Dias MWM 2
1 Secretaria Estadual de Saúde do Distrito Federal - Hospital Regional de Sobradinho.
2 Secretaria Estadual de Saúde do Distrito Federal - Hospital Regional Leste.

P-42. How old it’s too cold ? Live birth with sperm cryopreserved for 17 years.

Arent A. 1 2
Teloken C. 1
Teloken I. 1
Farinatti D. 1
Colombo T. 1
Azambuja R. 1
Petracco A. 1
Badalotti M. 1 2
1 FERTILITAT - Centro de Medicina Reprodutiva
2 Serviço de Ginecologia do Hospital São Lucas - PUCRS

P-43. Does embryo morphology in different maternal age groups interfere with the clinical pregnancy rates in frozen euploid blastocysts transfers?

Yoshida I.H. 1
Souto E. 1
Suganuma C.H. 1
Tanada M.S. 1
Berton C.Z. 1
Cordts E.B. 1 2
Barbosa C.P. 1 2
1 Instituto Ideia Fértil de Saúde Reprodutiva, Santo André/SP, Brasil.
2 Faculdade de Medicina do ABC/ Centro Universitário Saúde ABC, Santo André/ SP, Brasil.

P-44. Metformin: anovulation treatment and improvement in the reproductive profile of patients with polycystic ovarian syndrome

Baptista S.N.B. 1
Teixeira G.M. 1
Araujo N.G. 1
Pinheiro M.G. 1
1 Universidade Santo Amaro

P-45. Progestin-primed ovarian stimulation is an effective protocol for oocyte or embryo cryopreservation cycles: a cohort analysis and review of literature

Siqueira M.S. 1
Amaral A.S. 1
Amaral M.E. 1
Barbosa A.C.P. 1
Barbosa N.P. 1
Iglesias J.R. 1
Nakagawa H.M. 1
Barbosa M.W.P. 1
1 Genesis - Centro de Assistência em Reprodução Humana. Brasília-DF.

P-46. Profile of couples attended in a public-private reproduction center

Chiariello C.P. 1 2
Viana T.A. 1 2
Souza R.L.B. 1 2
Jayme J.C. 1 2
Labadessa L.M. 1 2
Bartmannn A.K. 1 2
1 Centro de Reprodução Humana Clinica Ana Bartmann – Ribeirão Preto - Brasil
2 Universidade de Ribeirão Preto – São Paulo /Brasil

